# TCA cycle enhancement and uptake of monomeric substrates support growth of marine *Roseobacter* at low temperature

**DOI:** 10.1038/s42003-022-03631-2

**Published:** 2022-07-14

**Authors:** Meng Wang, Huan Wang, Peng Wang, Hui-Hui Fu, Chun-Yang Li, Qi-Long Qin, Yantao Liang, Min Wang, Xiu-Lan Chen, Yu-Zhong Zhang, Weipeng Zhang

**Affiliations:** 1 College of Marine Life Sciences, Ocean University of China, Qingdao, 266003 China; 2 Frontiers Science Center for Deep Ocean Multispheres and Earth System, Ocean University of China, Qingdao, 266003 China; 3 State Key Laboratory of Microbial Technology, Marine Biotechnology Research Center, Shandong University, Qingdao, 266237 China; 4 Laboratory for Marine Biology and Biotechnology, Pilot National Laboratory for Marine Science and Technology, Qingdao, 266373 China

**Keywords:** Water microbiology, Bacterial genetics, Bacterial transcription

## Abstract

Members of the marine *Roseobacter* group are ubiquitous in global oceans, but their cold-adaptive strategies have barely been studied. Here, as represented by *Loktanella salsilacus* strains enriched in polar regions, we firstly characterized the metabolic features of a cold-adapted *Roseobacter* by multi-omics, enzyme activities, and carbon utilization procedures. Unlike in most cold-adapted microorganisms, the TCA cycle is enhanced by accumulating more enzyme molecules, whereas genes for thiosulfate oxidation, sulfate reduction, nitrate reduction, and urea metabolism are all expressed at lower abundance when *L. salsilacus* was growing at 5 °C in comparison with higher temperatures. Moreover, a carbon-source competition experiment has evidenced the preferential use of glucose rather than sucrose at low temperature. This selective utilization is likely to be controlled by the carbon source uptake and transformation steps, which also reflects an economic calculation balancing energy production and functional plasticity. These findings provide a mechanistic understanding of how a *Roseobacter* member and possibly others as well counteract polar constraints.

## Abstract (web summary)

The metabolic adaptation of *Loktanella salsilacus* strains to cold involves an increase of enzymes involved in the TCA cycle and preferential use of glucose rather than sucrose at low temperature, providing insights into how *Roseobacter* adapts in polar regions.

## Introduction

The potential habitats of cold-adapted microbes (bacteria and archaea) encompass a large proportion of the global biosphere and include diverse ecotypes such as polar regions, accounting for ~20% of the global surface; alpine areas, constituting ~5% of the continental surface; specific caverns; trenches of the deep sea; and some man-made biotopes^1^. Among these cold habitats, high-latitude oceans near Antarctica or Arctic Ocean foster the majority of previously isolated cold-adapted strains^2^. Prokaryotic organisms in polar waters have been demonstrated to play crucial roles in environmental processes and biogeochemical cycles, and they may generate an impact on global climate changes^3^.

Since the discovery of cold-adapted microbes, a great amount of effort has been put into deciphering their interactions with environmental factors. Temperature and dissolved organic material (DOM) are two major limiting factors for all marine heterotrophic microbes that live in polar regions. It was proposed that a ~80% fluctuation in the microbial growth rate in polar waters can be explained through the supply of labile DOM together with temperature^4^. Correspondingly, cold-adapted microbes have evolved diverse mechanisms to respond to these environmental stresses. Unlike heat shock protein, which is initiated by a major transduction factor (σ^32^ factor) to balance cellular homeostasis, no specific regulators for cold shock responses have been found^5^. However, based on the responses displayed by typical cold-adapted prokaryotes, such as *Colwellia psychrerythraea*^6,7^, *Planococcus halocryophilus*^8^, *Pseudoaltermonas haloplanktis*^9^, and *Psychrobacter* spp.^10,11^, several general mechanisms that appear to be important in cold-adapted microbes. These features are closely linked to the changes in membrane fluidity, the accumulation of anti-freeze proteins and compatible solutes (e.g., glycine, betaine, glycerol, and trehalose), the induction of cold-shock proteins (CSPs), and cold-adapted enzymes, the repression of reactive oxygen species (ROS), and the mediation of genome plasticity by horizontal gene transfer^12^. Besides temperature stress, different patterns of DOM incorporation also greatly influence heterotrophic bacterial growth in polar environments. *C. psychrerythraea* 34H, a typical psychrophile with widespread distribution in cold oceans, favors the use of the Entner–Doudoroff (ED) pathway rather than the Embden–Meyerhof–Parnas (EMP) pathway in the glycolytic route selection during cold acclimation^6^. This transformation provides a potential strategy for the simultaneous uptake of carbon sources and facilitates biomass production in nutrient-poor areas. Marine psychrotolerant *Vibrio* isolated from subarctic regions show a sensitive growth response at low temperature, depending on the concentration of available substrates^13,14^. These isolates exhibit an increasing requirement for organic nutrients at low temperature and their growth rates are significantly affected by substrate concentration near the minimum temperature for growth. In addition to the studies on cold-adapted strains, bacterial population analysis by fluorescence in situ hybridization (FISH) also demonstrates *Roseobacter* group bacteria (RGB) make up the majority of glucose-incorporating bacterial cells in the coastal North Sea waters^15^.

As an abundant heterotrophic bacterial group in marine ecosystems, RGB are defined as a subcluster of Rhodobacterales, sharing >89% identity in the 16S rRNA gene^16^. Although a reduced genome and low GC content are possessed by pelagic RGB members such as CHAB-I-5 strain SB2^17^ and *Planktomarina temperata* RCA23^18^, most members of the group have a large genome (average >4 Mbp) with a rich GC content (60% ± 4%) and are known for their metabolic diversity. These versatile capabilities mainly involve various mechanisms of obtaining energy, sulfur transformation, CO oxidation, aromatic compound degradation, and secondary metabolic production^16,19^. Moreover, the diverse lifestyles of this group mean that RGB bacteria can be free-living, particle-attached, or associated with marine phytoplankton and animals^20–22^. Benefiting from these characters, RGB members are distributed across a variety of marine habitats; they represent up to 20% of bacterioplankton phylotypes in specific environments of epipelagic oceans^23^ and show substantial abundance in polar habitats such as sea ice, springs, and ocean surface waters^24–26^. Due to their substantial abundance, highly active cells, and important roles in biochemical cycles, there is a critical need to explore the adaptations of RGB to the variability of marine environments.

Although a number of previous studies on cold-adapted microbes have been performed, the molecular mechanisms underlying the adaptations of RGB to cold environments are not well understood, largely due to a lack of a cold-adapted model organism. Moreover, the lack of integrated ecological and physiological studies of typical cold-adapted RGB members has hindered our understanding of the lifestyles of this microbial group. In the present study, we focused on two psychrotolerant RGB *Loktanella salsilacus* strains (1A07893 and 1A07899) isolated from Arctic regions and explored their cold-adaptive strategies by integrating genomics, metagenomics, transcriptomics, enzyme activity assays, and carbon utilization experiments. As the first published report of multi-omic information and cold-adapted characteristics of *L. salsilacus*, this article broadens our perspective of how RGB adapt to the polar ecosystem.

## Results

### Ability to thrive in cold environments

**Fig. 1:**
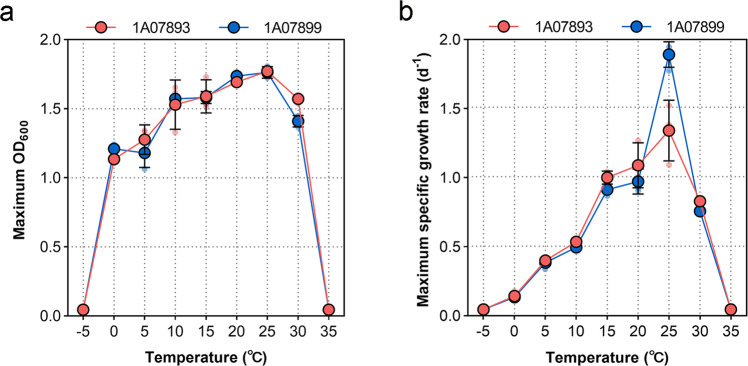
Growth of *L. salsilacus* at different temperatures. **a** Maximum growth biomass represented by OD_600_ of *L. salsilacus* 1A07893 and 1A07899 at temperatures ranging from −5 to 35 °C. **b** Maximum growth rates of 1A07893 and 1A07899 at temperatures ranging from −5 to 35 °C. Strains were grown in marine broth 2216 in triplicate. The error bars represent the standard deviation of three replicates.

The isolated locations of the two *L. salsilacus* strains are shown in Table 1. To investigate the cold-adaptive physiology of the two strains, their maximum biomass and maximum growth rates (*μ*_max_) represented by optical density at 600 nm (OD_600_) were monitored along a temperature gradient (Fig. 1a, b). Both strains were able to grow between 0 °C and 30 °C, and their optimal growth temperature (*T*_opt_) was 25 °C. Compared to the growth temperature profiles of cold-adapted RGB members reported in previous studies, *L. salsilacus* strains showed a lower minimum growth temperature than *Octadecabacter temperatus* SB1^27^ and *Octadecabacter antarcticus* 307^28^, and a lower *T*_opt_ than that of mesophilic RGB *Ruegeria pomeroyi* DSS-3^29^ and *Dinoroseobacter shibae* DFL 12^30^ (Supplementary Table 1). At the *T*_opt_, the maximum biomass represented by OD_600_ was 1.772 ± 0.034 for 1A07893 and 1.765 ± 0.043 for 1A07899, and the *μ*_max_ was 1.333 ± 0.219 d^−1^ for 1A07893 and 1.879 ± 0.092 d^−1^ for 1A07899. The *μ*_max_ values of *L. salsilacus* observed here were significantly lower than that of *D. shibae* DFL 12 and *Phaeobacter inhibens* DSM17395^31^ grown on the medium of similar components. The two strains were therefore considered as psychrotolerant bacteria according to the general definition of cold-adapted microbes^32^.

**Table 1:** Information of the two *L. salsilacus* strains applied in the present study.

Species	Strain	Location for isolation				Elevation (m)
		Area	Habitat	Longitude	Latitude	
*Loktanella salsilacus*	1A07893	Chukchi Sea	Sediment	167°02’ W	71°01’ N	−46
*Loktanella salsilacus*	1A07899	Chukchi Sea	Sediment	167°43’ W	71°49’ N	−50

**Fig. 2:**
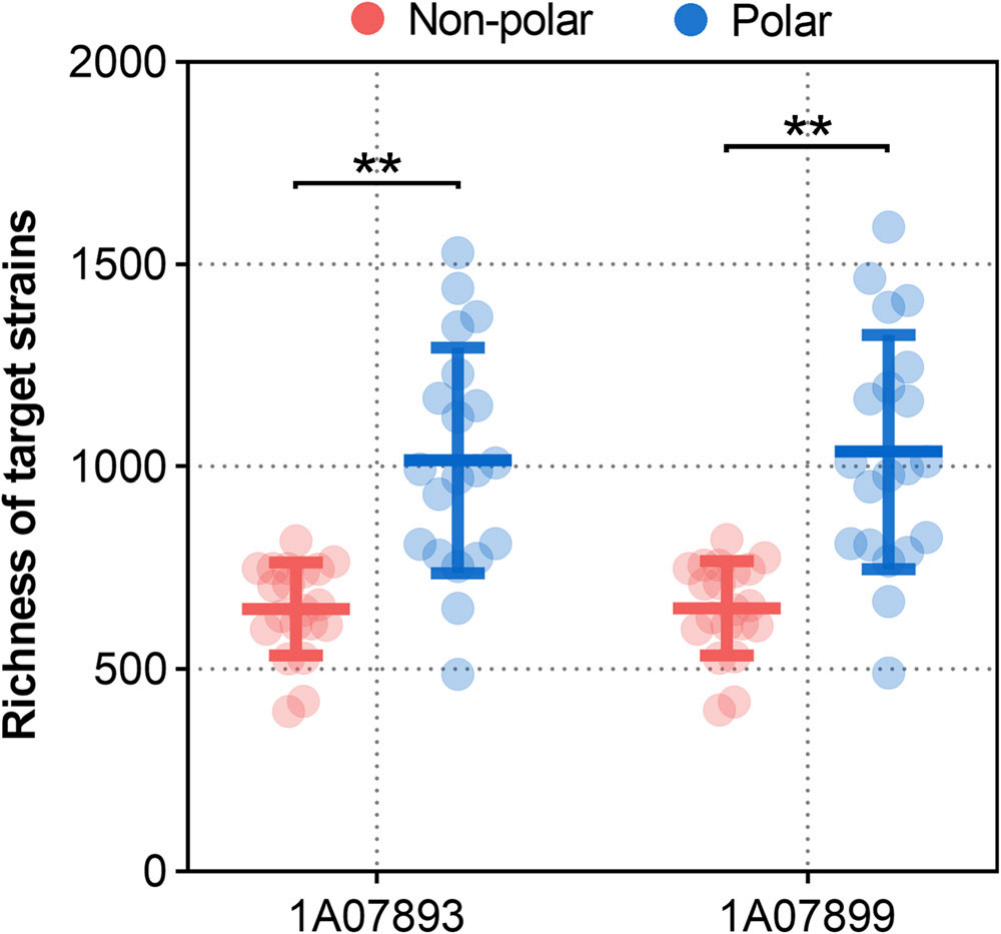
Global distribution of *L. salsilacus*. Abundances of *L. salsilacus* 1A07893 and 1A07899 recovered from 40 global seawater metagenomes, including those from polar and non-polar regions, are shown. Differences between the average abundance of *L. salsilacus* in non-polar and polar regions were estimated by a two-tailed *t*-test, and significant differences were indicated by asterisks (**p < 0.005).

In addition, to investigate the distribution patterns of *L. salsilacus* in natural environments, we scanned for their genomic sequences in 40 metagenomes that represent species from a range of global epipelagic seawaters, including those in polar and temperate regions. As a result (Fig. 2), we found the relative abundances of the two *L. salsilacus* strains were significantly higher in polar seawaters than non-polar regions (two-tailed *t*-test, *p* < 0.005). Strain 1A07893 and 1A07899 recruited more than 1‰ reads in 9 or 10 of the 20 polar seawater metagenomes, but both genomes recruited less than 1‰ reads in all non-polar seawater metagenomes (Supplementary Table 2). Compared to the recruited abundance of representative RGB strains (Supplementary Fig. 1), the higher richness of *L. salsilacus* in polar regions than that of mesophilic RGB was identified and a similar distribution pattern was also identified in psychrotolerant members *Octadecabacter* spp. and *Yoonia vestfoldensis* SMR4r. These results suggest that *L. salsilacus* is a typical cold-adapted member of the RGB that is highly enriched in polar regions.

### Monophyletic classification and heterotrophic genomic features

We first sequenced the complete genomes of the two *L. salsilacus* strains, and the general genomic features are shown in Supplementary Table 3. The 1A07893 genome consisted of a single chromosome of 3,250,852 bp with 3140 coding sequences (CDSs), a small plasmid of 138,781 bp with 139 CDSs, and a large plasmid of 597,811 bp with 539 CDSs; while the 1A07899 genome encompassed a chromosome of 3,401,541 bp with 3275 CDSs and a plasmid of 512,299 bp with 445 CDSs. GC skew analysis showed no detectable inversion or identical insertion (Supplementary Fig. 2).

**Fig. 3:**
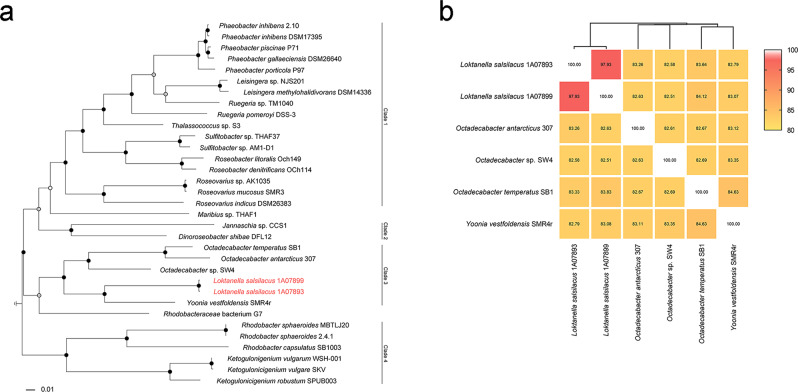
Evolutionary relationship between *L. salsilacus* and other representative RGB. **a** Phylogenetic tree based on the concatenation of 12 housekeeping genes (*dnaG*, *frr*, *infC*, *nusA*, *pgk*, *pyrG*, *rplC*, *rpmA*, *rpoB*, *rpsC*, *smpB*, and *tsf*). Bootstrap values generated by 1000 bootstraps were labeled at nodes (white, ≥35%; gray, ≥50%; black, ≥75%). **b** Pairwise comparisons between *L. salsilacus* and closely related strains to reveal average nucleotide identity (ANI) values. ANI values were labeled in corresponding cells of the heatmap, and phylogenetic distances were calculated by a complete clustering algorithm based on Euclidean geometry.

A phylogenetic tree based on a concatenation of 12 conserved genes of *L. salsilacus* and representative RGB strains differentiated into four major lineages (Fig. 3a), and the topology was overall consistent with our previous understanding of these genera^17,19^. *L. salsilacus* formed an independent branch within Lineage 3 to establish monophyly within the RGB, which was supported by a bootstrap value of 97% and could be recovered repeatedly with maximum likelihood calculations. Lineage 3 also contained *O. temperatus* SB1 from the North Sea^27^, *O. antarcticus* 307 isolated from the Antarctica^28^, *Octadecabacter* sp. SW4 from Russia, and *Y. vestfoldensis* SMR4r from Oresund, Sweden^33^, and thus, most members of this lineage were isolated from regions associated with the Arctic/Antarctic circles. Moreover, a whole-genome comparison based on the average nucleotide identity (ANI) was performed (Fig. 3b), which further supported the classification of 1A07893 and 1A07899 as the same species, as the ANI value was above the species differentiation cutoff (i.e., 95%)^34^.

The genomic variations between 1A07893 and 1A07899 were also identified (Supplementary Fig. 3). According to the annotation results of Kyoto Encyclopedia of Genes and Genomes (KEGG), the two genomes shared 1879 KEGGs, whereas only 118 and 94 KEGGs were specific for 1A07893 and 1A07899, respectively (Supplementary Fig. 3a). The KEGGs common between the two genomes generally belonged to the categories of carbohydrate metabolism, amino acid metabolism, and energy metabolism (Supplementary Fig. 3b). The major differences between the two genomes were related to the type of bacterial secretion system: strain 1A07893 carried the type IV secretion system, whereas 1A07899 carried the type VI secretion system (Supplementary Fig. 3c).

We next performed comparative genomic analyses of the two *L. salsilacus* strains and four selected RGB strains as references (Supplementary Fig. 4). Our results demonstrated that the trophic strategy of *L. salsilacus* appears to be typical of chemoheterotrophs, and no light-harvesting genes, including the bacteriochlorophyll α synthesized by aerobic anoxygenic phototrophs or rhodopsin, were detected in the genomes. Moreover, carbon-fixation genes, such as ribulose-bisphosphate carboxylase (*rbcL*) and pyruvate ferredoxin oxidoreductase (*porB*) found in *O. antarcticus* 307 and 2-oxoacid ferredoxin oxidoreductase (*korB*) in *Y. vestfoldensis* SMR4r, were absent in the strains. The complete tricarboxylic acid (TCA) cycle, ED pathway, and pentose phosphate (PP) pathway were present in 1A07893 and 1A07899; whereas 6-phosphofructokinase, a key enzyme of the EMP pathway encoded by *pfkB*, was absent, suggesting that the flux of carbohydrates fed into the TCA cycle is mainly captured by the ED or PP pathway, rather than the EMP pathway. Demonstrated by *P. inhibens* DSM 17395 previously^35^, the use of the ED or PP pathway due to the absence of 6-phosphofructokinase in the EMP pathway is an important physiological property among heterotrophic marine bacteria. The phosphate acetyltransferase-acetate kinase pathway was also reconstructed in both *L. salsilacus* strains; this pathway has been demonstrated to control the level of acetyl phosphate and to play a role in regulating cell motility, chemotaxis, biofilm formation, and temperature stress^36,37^.

To facilitate energy generation through the oxidation of inorganic matter (Supplementary Fig. 4), *L. salsilacus* carries a Sox system (*soxAB*, *soxYZ*), which oxidizes thiosulfate into sulfate. Other sulfur and nitrogen metabolism genes, such as phosphoadenosine phosphosulfate reductase (*cysH*) for sulfate reduction, nitrate reductase (*nasA*), and nitrite reductases (*nirB*, *nirD*) for nitrate reduction, were also present. In addition, genes for metabolizing the ubiquitous marine organic compounds dimethylsulfoniopropionate and trimethylamine, such as dimethylsulfoniopropionate demethylase (*dmdA*), dimethylpropiothetin dethiomethylase (*dddL*) and trimethylamine monooxygenase (*tmm*), were totally absent from the two *L. salsilacus* genomes (Supplementary Fig. 4).

### Low-temperature-induced transcription of TCA and respiratory chain genes

**Fig. 4:**
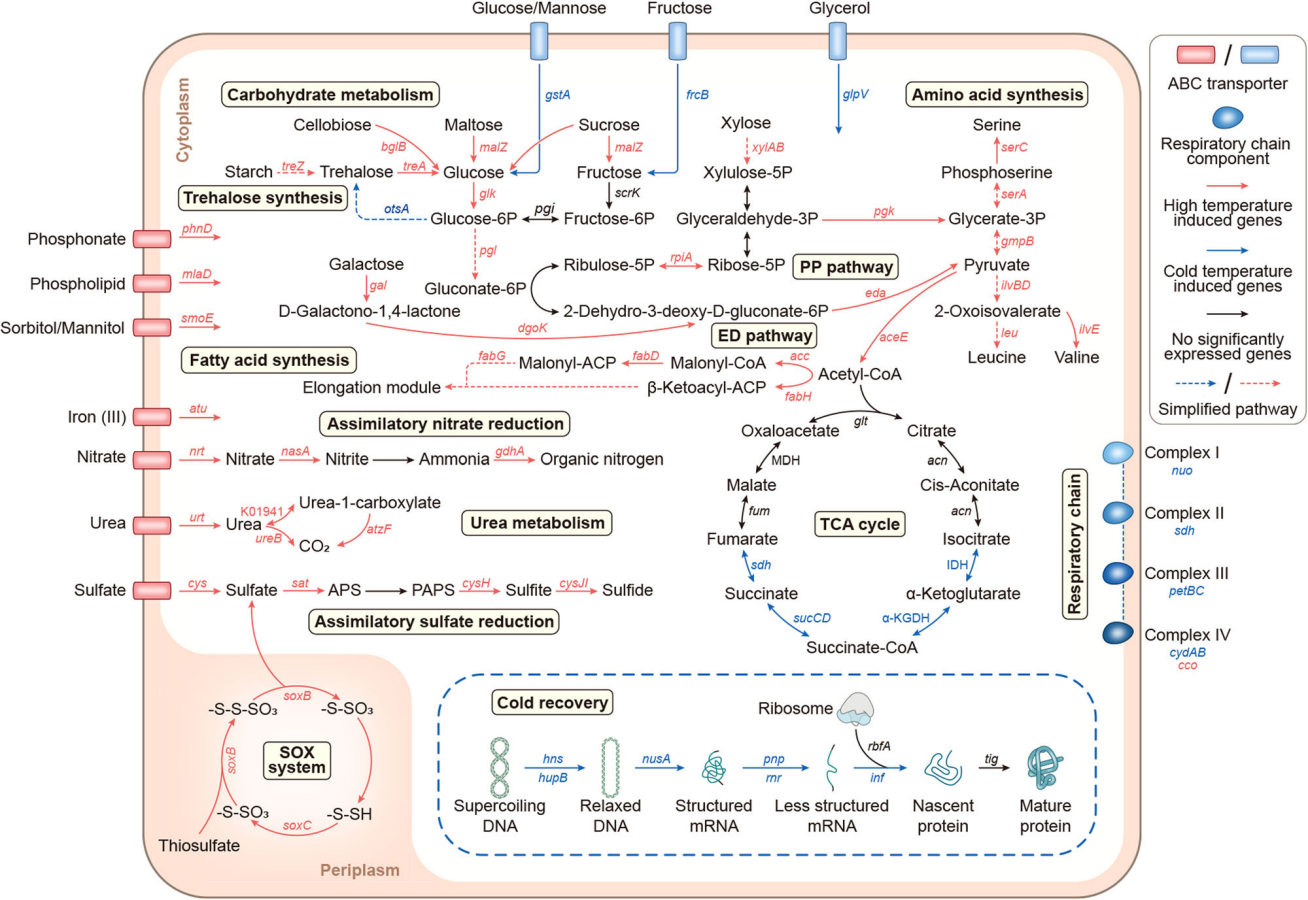
Differentially transcribed genes and pathways in *L. salsilacus* with temperature variation. Reads per kilobase per million mapped reads (RPKMs) generated from *n* = 3 biologically independent samples of each group were used to indicate the gene transcriptions of *L. salsilacus* grown in marine broth 2216. The significance of each difference was judged by the absolute value of log_2_ (fold-change of RPKMs) >1 and *p*-value <0.05. Differentially expressed genes associated with the temperature in *L. salsilacus* were concluded when the number of genes obtained from the pairwise comparisons between different temperatures was ≥2. Low-temperature-induced genes and corresponding reactions were labeled with blue, while those that were moderate-temperate-induced are labeled with red. Simplified pathways were drawn with dashed lines.

To obtain information on gene-level mechanisms that facilitate cold adaptation by *L. salsilacus*, gene transcription profiles of cultures grown in marine broth were studied at five temperatures (5, 10, 15, 20, and 25 °C), and the temperature-dependent metabolic profiles were reconstructed based on differentially transcribed genes (Fig. 4 and Supplementary Fig. 5). First, a series of CSPs, including the nucleoid-associated proteins HUβ (*hupB*) and H-NS (*hns*), transcription termination/antitermination protein (*nusA*), 3′ to 5′ exoribonuclease RNase R (*rnr*), and PNPase (*pnp*), showed significantly higher transcription levels at 5 °C. Notably, cold temperature induced the transcription of many genes belonging to the TCA cycle, including isocitrate dehydrogenase (IDH), α-ketoglutarate dehydrogenase (α-KGDH), succinyl-CoA synthetase (*sucCD*), and succinate dehydrogenase (*sdhABCD*), in both strains. Furthermore, respiratory chain components, including NADH dehydrogenase (*nuoABC*), cytochrome c reductase (*petBC*), and bd-type cytochrome c oxidases (*cydAB*), also showed higher transcription levels at low temperature. Regarding transporter-encoding genes, monosaccharide transporters, such as glucose transporter (*gstA*) and fructose transporter (*frcB*), were upregulated at 5 °C. Additionally, the upregulation of the glycerol transporter gene (*glpV*) and the trehalose-6-phosphate synthase (*otsA*) suggested the accumulation of intracellular compatible solutes may be essential for *L. salsilacus* adaptation to cold. To summarize, low temperature tends to induce the transcription of genes related to cold stress responses, energy metabolism, and the uptake of simple carbohydrates.

By contrast, genes encoding proteins involved in other metabolic pathways, such as glycolytic routes, fatty acid biosynthesis, amino acid synthesis, thiosulfate oxidation (Sox system), sulfate/nitrate reduction, and urea metabolism, were depressed at 5 °C (Fig. 4 and Supplementary Fig. 5). For example, most of the genes involved in amino acid metabolism, including *serAC*, *ilvBED*, and *leuBCD*, were upregulated with an increase in temperature. Most genes for proteins involved in the metabolism of nitrogen and sulfur compounds, such as *nasA*, *cysHJI*, and *soxBC*, were also upregulated at moderate to high temperature. Moreover, transmembrane transporters related to the uptake of phosphate-related materials (*phnD*), polyol and sugar alcohol (*mlaD*, *smoE*), Fe^3+^ (*afu*), nitrogen (*nrtABC*, *urtACE*), and sulfate sources (*cysPU*) were depressed at cold temperature.

### Validation of the transcriptomic results by RT-qPCR and growth test

To validate the transcriptomic results, we performed RT-qPCR and a growth experiment using citric acid or succinate as the sole carbon source. The quantitative results of the RT-qPCR showed consistency with transcriptomic data. For strain 1A07893, a respiratory chain gene (*cydA*) and three genes of the TCA cycle (*sucC*, IDH, and α-KGDH) were upregulated at 5 °C (Supplementary Fig. 6a) in comparison with 25 °C. Similarly, the transcription levels observed for *nuoA*, *cydA*, *sdhB*, IDH, and α-KGDH in strain 1A07899 were significantly higher at 5 °C than 25 °C (Supplementary Fig. 6b).

In addition, the maximum biomass represented by OD_600_ of 1A07893 grown on citric acid was 0.828 ± 0.025 at 25 °C in comparison with 1.013 ± 0.003 at 5 °C, and on succinate was 0.480 ± 0.001 at 25 °C in comparison with 0.691 ± 0.009 at 5 °C (Supplementary Fig. 6c). A similar result was also obtained with strain 1A07899. Hence, cold temperature induced a ~13% increase in the biomass of *L. salsilacus* when grown on intermediates of the TCA cycle, which was a significant increase compared with the biomass at room temperature.

### Accumulation of more TCA enzyme molecules at low temperature

**Fig. 5:**
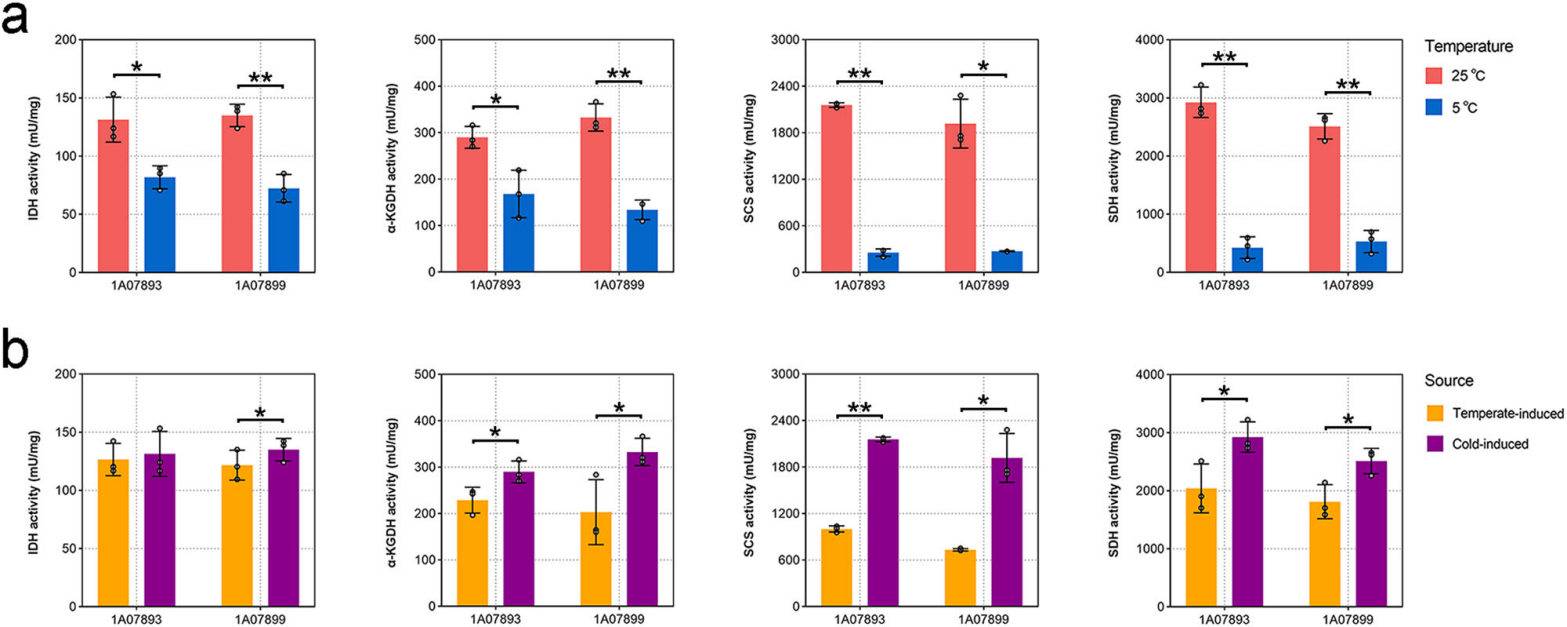
Activities of the TCA cycle enzymes. **a** Activities of low-temperature-induced enzymes extracted from cultures at 5 °C and measured at 5 and 25 °C. **b** Activities of cold-induced (derived from cultures at 5 °C) and temperate-induced (derived from cultures at 25 °C) enzymes measured at 25 °C. One unit of IDH was defined as the amount of enzyme that generates 1.0 μmole of NADPH per minute at pH 8.0 at 5 °C or 25 °C, one unit of α-KGDH was defined as the amount of enzyme that generates 1.0 μmole of NADH per minute at pH 7.5 at 5 °C or 25 °C, one unit of SCS was defined as the amount of enzyme that generates 1.0 mmole of NADH per minute at pH 7.4 at 5 °C or 25 °C, and one unit of SDH was defined as the amount of enzyme that generates 1.0 μmole of DCIP per minute at pH 7.2 at 5 °C or 25 °C. The experiments were performed in triplicate and the average values are given. Error bars represent standard deviations. Significant differences between the two datasets are indicated by asterisks (*p < 0.05; **p < 0.005).

Based on the transcriptomic results, we hypothesized that the enhanced TCA cycle with a respiratory chain may play a key role in *L. salsilacus* energy generation during cold adaptation. To examine this hypothesis, the activities of IDH, α-KGDH, SCS, and SDH, key enzymes of the TCA cycle, were measured. First, the activities of cold-induced enzymes extracted from cultures grown at 5 °C were measured at 5 °C and 25 °C (Fig. 5a). The absolute activities of IDH, α-KGDH, SCS and SDH from 1A07893 were 131.444 ± 19.321, 290.094 ± 23.505, 2159.790 ± 29.927 and 2926.585 ± 260.782 mU/mg at 25 °C, compared with 81.854 ± 9.830, 168.227 ± 51.200, 255.248 ± 47.611 and 424.933 ± 187.991 mU/mg at 5 °C. Similarly, the absolute activities of IDH, α-KGDH, SCS, and SDH from 1A07899 grown at 5 °C showed significantly higher enzymatic activity when measured at 25 °C than 5 °C. Next, the activities of cold-induced (derived from cultures at 5 °C) and temperate-induced (derived from cultures at 25 °C) IDH, α-KGDH, SCS, and SDH were compared at 25 °C (Fig. 5b). For 1A07899, the activities of temperate-induced IDH, α-KGDH, SCS and SDH were 121.708 ± 12.823, 203.066 ± 70.117, 734.239 ± 14.963 and 1812.168 ± 292.504 mU/mg, respectively, significantly lower than the cold-induced enzymes: IDH, 135.096 ± 9.660 mU/mg, α-KGDH, 332.845 ± 29.448 mU/mg, SCS, 1917.894 ± 315.299, and SDH, 2513.225 ± 218.554 mU/mg. Although no significant change in the activity of IDH from 1A07893 was detected, similar results were obtained for the activities of α-KGDH, SCS, and SDH. Therefore, the absolute enzymatic activities of IDH, α-KGDH, SCS, and SDH on a per-molecule basis were not enhanced by low temperature, but their activities on a per-cell basis were increased when the bacterial strains were grown at low temperature. It is likely that *L. salsilacus* accumulates more copies of the TCA enzymes at low temperature by regulating gene transcription.

### Preferential utilization of monosaccharides at low temperature

Substrate selection is correlated with carbon assimilation efficiency and growth performance for RGB^38^. To study the selective utilization of carbon sources at different temperatures, 1A07893 and 1A07899 were grown in media with different carbohydrates as the sole carbon source. These carbon sources included monosaccharides that are common in the Arctic surface water column (glucose, fructose, galactose, rhamnose, and xylose)^39–41^ and different classes of their polymers, including disaccharides (sucrose, maltose, and cellobiose) and polysaccharides (starch and xylan). We found that both strains could use glucose, fructose, galactose, xylose, sucrose, maltose, or cellobiose as the sole carbon source. Except when 1A07893 used fructose as the sole carbon source, the strains grown on monosaccharides accumulated significantly (*p* < 0.05) higher biomass at 5 °C than those grown at 25 °C (Supplementary Fig. 7). By contrast, strains grown on disaccharides or polysaccharides, without 1A07899 grown on cellobiose, accumulated significantly (*p* < 0.05) lower biomass at 5 °C than those grown at 25 °C. For instance, when strain 1A07899 was grown on glucose, the maximum biomass was 0.551 ± 0.017 at 25 °C, which was significantly lower than 0.727 ± 0.012 at 5 °C, but when incubated in sucrose, the biomass reached 0.559 ± 0.003 at 25 °C, which was significantly higher than 0.453 ± 0.001 at 5 °C. The biases were also seen among the other carbon sources available to each strain.

**Fig. 6:**
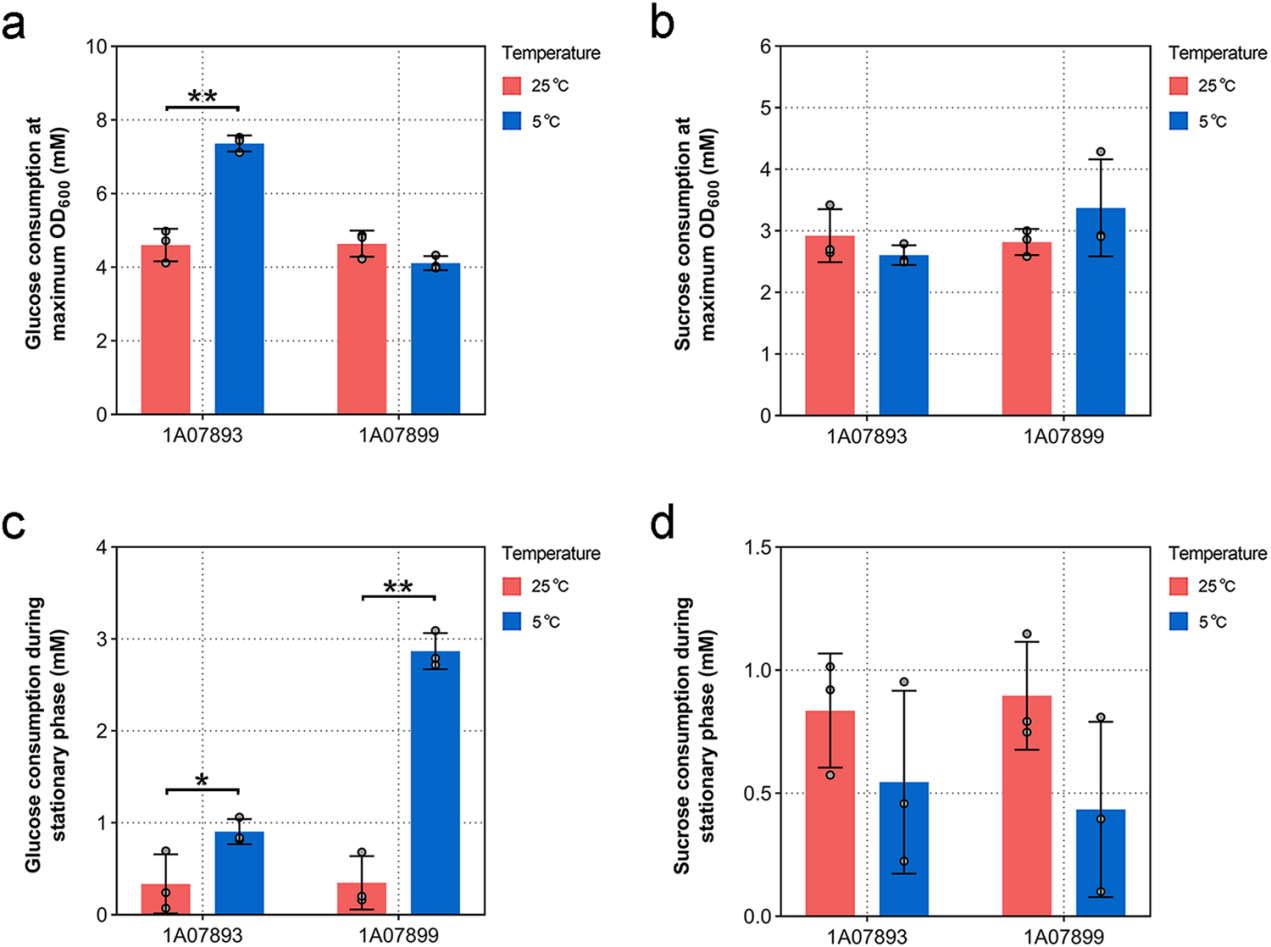
Competitive carbon consumption experiments comparing the utilization of glucose and sucrose by *L. salsilacus*. **a** Glucose consumed by *L. salsilacus* grown on the defined substrate mixture were measured at maximum OD_600_ at 5 °C and 25 °C. **b** Sucrose consumed by *L. salsilacus* grown on the defined substrate mixture were measured at maximum OD_600_ at 5 and 25 °C. **c** Glucose consumed by *L. salsilacus* incubated in the defined substrate mixture were measured during the same time in stationary phase. **d** Sucrose consumed by *L. salsilacus* incubated in the defined substrate mixture were measured during the same time in stationary phase. The experiments were performed in triplicate and the average values are given. Error bars represent standard deviations, and significant differences are indicated by asterisks (*p < 0.05; **p < 0.005).

To further support the results of the selective utilization of carbon sources at different temperatures, a carbon source competition experiment of *L. salsilacus* grown on a defined substrate mixture was performed. The two *L. salsilacus* strains were grown on the media containing glucose and sucrose at equal concentrations (10 mM) at 5 and 25 °C, and the consumption of glucose (to represent monosaccharides) and sucrose (to represent disaccharides) through two stages were monitored: 1) from the initial cultivation to the maximum OD_600_ and 2) from the maximum OD_600_ to the end of cultivation (i.e., the stationary phase). The maximum biomass represented by OD_600_ of 1A07893 was 0.687 ± 0.038 at 25 °C and 0.740 ± 0.044 at 5 °C (Supplementary Fig. 8a), and 1A07899 was 0.561 ± 0.021 at 25 °C and 0.509 ± 0.019 at 5 °C (Supplementary Fig. 8b). Therefore, *L. salsilacus* grown on the defined substrate mixture attained similar biomass at different temperatures. During the growth period, strains’ preference for glucose was indicated by the fact that more glucose than sucrose was consumed at both temperatures, but the preference was significantly enhanced during cold acclimation (Supplementary Fig. 8c, d). In detail, during the first growth stage of 1A07893, the consumption of glucose was 4.606 ± 0.440 mM at 25 °C, which was significantly (*p* < 0.005) lower than 7.365 ± 0.215 mM at 5 °C (Fig. 6a). No significant differences were found in the consumption of sucrose between 5 and 25 °C (Fig. 6b). Meanwhile, the temperature showed no significant influence on glucose or sucrose consumption of 1A07899. During the second growth stage of 1A07893, the consumption of glucose after the same time was 0.905 ± 0.136 mM at 5 °C and it was significantly (*p* < 0.005) higher than 0.336 ± 0.322 mM at 25 °C (Fig. 6c), whereas no significant changes in sucrose consumption were observed between 5 and 25 °C (Fig. 6d). A similar utilizing pattern of glucose and sucrose consumed by 1A07899 at different temperatures also have been identified in the stationary growth stage.

## Discussion

Ecological factors, such as substrate and temperature are general limiting characteristics of cold environments. Of these, substrate and temperature exert an appreciable regulating effect on bacterial growth in polar waters. Microbes import carbon sources from external environments, transform them via intracellular metabolic cycles, and produce energy via respiratory apparatus. Thus, controlling these steps appropriately is likely to be important for microbial adaptation to certain environments. In the present study, as a representative of cold-adapted RGB, cold adaptive mechanisms of two *L. salsilacus* strains were characterized. Through physiological experiments and bioinformatic analyses, we identified the psychrotolerant feature of *L. salsilacus* and its widespread distribution in the Arctic regions, in line with previous findings that the genus *Loktanella* is a major RGB lineage in the Arctic^25,42^. More importantly, our results demonstrate that the enhanced TCA cycle plays an important role in energy generation during cold acclimation, which is accompanied by the selection of the glycolytic pathway and the induction of a set of CSPs. The cold adaptation mechanisms of *L. salsilacus* are discussed in the following.

Based on genomic analyses, *L. salsilacus* strains appear to be typical chemoheterotrophs and totally rely on the metabolism of organic carbons via central carbon metabolism pathways represented by glycolytic pathways and the TCA cycle. The ED and PP pathway are likely to play crucial roles in *L. salsilacus* carbohydrate metabolism, as the key enzyme (phosphofructokinase) of the EMP route is entirely absent. The bypassing of the EMP pathway was also demonstrated by proteomics of *P. inhibens* DSM 17395^35^ and ^13^C flux profiling of *Loktanella* sp. D3^43^. Even in *C. psychrerythraea* 34H where all three glycolytic pathways coexist, advanced flux through the ED pathway instead of the EMP pathway was observed when the culture was grown at 4 °C^6^. This strategy would be a tradeoff between energy production and the cost of protein biosynthesis, as the EMP pathway produces higher amounts of ATP but requires more enzymatic proteins to catalyze pathway flux^44^. Moreover, due to the increased concentration of reactive oxygen species (ROS) at low temperature, the intracellular NAD(P)H from the ED or PP pathway could be used as a major redox currency in the elimination of ROS damage^45,46^.

The transcriptional upregulation of glycolytic routes at high temperature could be explained by their roles in generating precursor metabolites for compounds biosynthesis. In *L. salsilacus*, ED and PP pathways are favored when the cells require protein and/or lipids synthesis at high temperature while depressed along with synthesis demand at low temperature. However, as the other key component of central carbon metabolism, the TCA cycle shows a reverse transcriptional pattern to the temperature gradient. The inconsistent transcript profiles in the two components of central carbon metabolism may explain by different applications of glycolytic pathways when *L. salsilacus* are grown at different temperatures. As a key intermediate of glycolytic pathways, pyruvate derived from ED and PP pathways mainly convert to acetyl-CoA through oxidative decarboxylation and amino acids in aerobic and high temperature conditions. Due to the accumulated transcripts of acetyl-CoA carboxylase (*acc*), acyl carrier protein (ACP) synthase (*fabH*) and ACP malonyltransferase (*fabD*), mainly acetyl-CoA is then transformed and employed as a precursor to bacterial fatty acid biosynthesis. During cold acclimation, ED and PP pathways are depressed together with the biosynthesis routes, but the transcripts of citrate synthase still maintain the same level as that at high temperature. Therefore, we propose that glycolytic pathways mainly favor protein and/or lipids synthesis at high temperature while support energy generation by linking the TCA cycle during cold acclimation.

The transcriptional up-regulation of the TCA cycle and oxidative phosphorylation could be related to energy generation metabolism. At low temperature, *L. salsilacus* upregulates the transcription of key enzymes of the TCA cycle and oxidative phosphorylation to provide energy, as these are the most efficient way of energy production, especially in environments where molecular oxygen is available as a terminal electron acceptor. However, the last step of the TCA cycle, when L-malate is dehydrogenated to oxaloacetate and NAD^+^ is reduced to NADH, and the last step of oxidative phosphorylation, when ATP is synthesized from the proton gradient between cellular membranes, are not upregulated. We assume that this precise regulation is related to thermodynamic considerations, as substrate concentrations below an enzymes’ Michaelis constant (Km) will slow down the reaction. From the enzyme activity perspective, our results show that low temperature induces *L. salsilacus* to synthesize more TCA enzyme molecules, rather than change the absolute activity of the enzymes. To compensate for this cost of protein synthesis, *L. salsilacus* chooses to down-regulate genes responsible for several metabolic reactions, which further leads to the selective utilization of carbon sources at low temperature.

In addition, we demonstrate that the preferential utilization of monosaccharides by *L. salsilacus* is enhanced by low temperature. The influence of temperature on bacterial carbon utilization has been uncovered in previous studies. For instance, as the temperature increased from 13 to 45 °C, there was a 50% decrease in carbon-source utilization by bacteria isolated from caves in northern Spain^47^. *Campylobacter jejuni* 11168, colonizing in the intestinal tracts of avian species, can utilize a variety of carbon sources, such as amino acids, L-serine, L-aspartic acid, L-asparagine, and L-glutamic acid. The oxidation of these substrates is generally greater at 42 than 37 °C^48^. Psychrophilic *Vibrio* sp. AF-1 produces the maximum cell yield when grown on glucose and lactose at temperature <8 °C, which is probably attributed to the higher substrate affinities for these monosaccharides at temperature <8 °C than at temperature >15 °C^49^. In the present study, the preferential utilization of monosaccharides by *L. salsilacus* grown at low temperature has been demonstrated by the growth advantages attained from cultures growing in a sole carbon source medium at 5 °C. Meanwhile, when *L. salsilacus* were grown at a defined mixture of substrates containing equal amounts of glucose and sucrose, the preferential utilization of glucose is favored at both 5 and 25 °C, in comparison with sucrose. However, the preferential utilization of glucose is more popular at 5 °C than at 25 °C. All these results point to a correlation between low temperature and the preferential utilization of monosaccharides by *L. salsilacus*, which is probably controlled by both uptake and transformation steps. The uptake of carbon sources is primarily mediated by ATP-binding cassette (ABC) transporters, which are transmembrane proteins that utilize the energy of ATP hydrolysis to carry out certain transmembrane translocation of various substrates including sugars, amino acids, lipids, ions, and peptides. The upregulated ABC transporters for glucose during cold acclimation may support strains’ high affinity for this substrate even its extracellular concentration is rare. Moreover, high-molecular-weight nutrients often need to be broken down before flowing into central carbon metabolism for energy generation or metabolites synthesis, which needs the additional synthesis of intracellular or extracellular enzymes and more ATP. Thus, non-monomeric nutrients like sucrose may bear more energy, but the utilization of these nutrients from external environments may not be economical and tightly regulated by cold-adapted microbes. Although carbohydrates may not be the major carbon source or energy source for prokaryotes in natural environments, their crucial roles in sustaining bacterial growth have been identified by the RGB’s priority for carbohydrates even when sugar is not the main substrate in the defined medium^38^. Hence, the pattern of *L. salsilacus* in carbohydrates utilization explained in this study is meaningful for the understanding of the typical metabolic characteristics of cold-adapted RGB.

To counteract cold stress, *L. salsilacus* also induce a specific set of CSPs, facilitating transcription and translation. The mechanisms by which these CSPs favor cold acclimation have been clarified, e.g., Huβ is involved in the modification of supercoiling DNA and allows new initiation events at *oriC*^50^, RNaseR is involved in the quality control of rRNAs by eliminating RNA secondary structure^5^, and the family of initiation factors promotes the initiation of translation^51–53^. Biosynthetic pathways, such as amino acid biosynthesis, assimilatory sulfate, and nitrate reduction, are also down-regulated, consistent with the lower proliferation rate displayed when *L. salsilacus* were grown in marine broth at low temperature. The consistent up-regulation of these CSPs was also validated by our transcriptome sequencing data.

To summarize, the present study has illustrated that *L. salsilacus* is a representative cluster of the RGB in cold environments and that the enhanced TCA cycle and preferential utilization of monosaccharides play crucial roles in the cold adaptation of this species. These findings have promoted our understanding of the strategies by which marine RGB overcome polar constraints. In the future, the catabolism of amino acids and other carbon sources in psychrotolerant RGB would be studied, as well as the relationship between carbon assimilation efficiency and biomass accumulation rate. In addition, the exploration of cold-adapted RGB in polar sediments, bearing a variety of cold-adapted prokaryotes, is hindered by the lacking of well-constructed metagenomic datasets, which warrants further investigation.

## Methods

### Bacterial strains and growth conditions

Two strains of *L. salsilacus* isolated from the Arctic Ocean were obtained from the Marine Culture Collection of China (MCCC, https://mccc.org.cn), and the detailed information is given in Table 1. Purified strains were precultured at 25 °C, 180 rpm, in marine broth 2216 (Difco, Franklin Lakes, NJ, USA) and used as inoculum for the following procedures. The growth performances were validated at different temperatures (−5 to 35 °C) in marine broth 2216 in triplicate, and the cell concentrations were monitored based on OD_600_ with a spectrophotometer (T6, Persee Analyticals, Beijing, China). The maximum specific growth rate of each temperature was calculated on DMfit v3.5 using the Gompertz model (https://www.combase.cc).

### Genome sequencing and assembly

The genomic DNA of the tested strains was extracted via the SDS method^54^. The harvested DNA was detected by agarose gel electrophoresis and quantified with a Qubit 2.0 Fluorometer (Thermo Scientific, MA, USA). The whole genome of each strain was sequenced using a PacBio Sequel platform and Illumina NovaSeq PE150 at Beijing Novogene Bioinformatics. After data generation, the low-quality reads of PacBio (less than 500 bp) were filtered to obtain clean data. Preliminary assembly was performed with SMRT Link v5.0.1 (https://www.pacb.com/support/software-downloads), then corrected by both arrow algorithm and Illumina data using bwa. Finally, the chromosome and plasmid sequences were screened by BLAST to form a complete genome. The circular plot of the complete genome of each *L. salsilacus* strain was generated by CGView v1.0^55^.

### Assessment of *L. salsilacus* cluster in global prokaryotic communities

Prokaryote-enriched metagenomes from non-polar regions of the Tara Ocean (*n* = 20) and Arctic Ocean (*n* = 20) (sampling station information is supplied in Supplementary Table 2) were used to investigate the distribution of each *L. salsilacus* strain in the global oceans^56,57^. For each metagenome, low-quality reads were first filtered by the NGS QC Toolkit v2.3.3 with default parameters and converted to FASTA format^58^. One million reads were then extracted randomly from each dataset by SeqKit v0.15.0^59^ and applied to measure the abundance of *L. salsilacus* using BBMap v38.87 (https://sourceforge.net/projects/bbmap) for each station. Differences between the average abundance of *L. salsilacus* in non-polar and polar regions were estimated by a two-tailed *t*-test, and significant differences were concluded when the *p*-value was <0.05.

### Genome annotation and phylogenetic analyses

Aside from the two genomes of *L. salsilacus*, another 31 complete genomes representing RGB were collected for this study (Supplementary Table 4). For all strains, the protein-coding genes were predicted using Prokka v1.14.6 with default parameters^60^, and protein sequences of tested strains were then used in BLASTP analysis (*E*-value <1e−7) against the KEGG database^61^. The pathway was identified when over half of the related genes were recruited without the exclusion of key proteins.

To generate the phylogenetic tree, 12 housekeeping genes (*dnaG*, *frr*, *infC*, *nusA*, *pgk*, *pyrG*, *rplC*, *rpmA*, *rpoB*, *rpsC*, *smpB*, and *tsf*) were extracted from 31 representative RGB strains and two *L. salsilacus* strains using AMPHORA2^62–64^. The ClustalW alignment and maximum likelihood tree based on concatenated housekeeping genes were generated in MEGA v6.06^65^, and visualized by ggtree v3.12 with 1000 bootstrap values^66^.

Pairwise genome comparisons of the two *L. salsilacus* strains, *O. antarcticus* 307, *Octadecabacter* sp. SW4, *O. temperatus* SB1, and *Y. vestfoldensis* SMR4r were conducted with ANI using the ANI Calculator of EZBioCloud^67^, and the ANI heatmap was created using GraphPad Prism v8.0. The distance was calculated using a complete clustering algorithm based on Euclidean geometry.

### Transcriptome sequencing and analyses

*L. salsilacus* strains were cultured in triplicate in marine broth 2216 at five different temperatures (5, 10, 15, 20, and 25 °C) to the early stationary phase. The cells at each temperature were harvested at 5000 × *g* for 5 min and immediately transferred into liquid nitrogen. Total RNA was extracted from each replicate using RNAprep Pure Cell/Bacteria Kit (Tiangen Biotech, Beijing, China) to construct RNA-seq libraries using the NEBNext Ultra RNA Library Prep Kit for Illumina (New England Biolabs, USA) following the manufacturer’s recommendations. The library preparations were then sequenced on an Illumina Novaseq platform at Beijing Novogene Bioinformatics, and 150 bp paired-end reads were generated. Raw transcriptome data were quality-controlled using the NGS QC Toolkit v2.3.3, the index of each *L. salsilacus* genome was built, and clean reads were mapped to the corresponding genome using Bowtie 2 v2.4.2^68^. To standardize the genetic expression, the number of reads mapped to each gene from transcriptomes was converted to reads per kilobase per million mapped reads (RPKMs) using Samtools v1.11^69^ and an in-house perl script. The differences were considered significant when the *p*-value was <0.05 and the absolute value of log_2_ (fold-change) of RPKMs was >1. Differentially expressed genes associated with temperature in *L. salsilacus* were concluded when the number of genes obtained from the pairwise comparisons between different temperatures was ≥2. The statistical enrichment of these genes in KEGG pathways was analyzed by KEGG Mapper (https://www.genome.jp/kegg/mapper.html).

### RT-qPCR assays

Respiratory chain- (*nuoA*, *cydA*) and TCA- (*sucC*, *sdhB*, IDH, α-KGDH) related transcripts were selected to test the results of the RNA-seq analyses. RNA samples were extracted from cell cultures of each strain grown to the early stationary phase at 5 °C or 25 °C using the RNeasy Mini Kit (Qiagen, Dusseldorf, Germany). All-in-One First-Strand cDNA Synthesis SuperMix for qPCR (Transgen, Beijing, China) was used to convert RNA into random-primed cDNA according to the manufacturer’s protocol. The primer pairs (Supplementary Table 5) were designed and validated by BLAST to generate a single-peak dissociation curve for each gene, and the housekeeping gene *rplC* was selected as the internal reference. The RT-qPCR reactions were performed with triplicates for each RNA extract on QuantStudio 6 Flex (Thermo Scientific) using the SYBR Green Premix Pro Taq HS qPCR Kit (Accurate Biotechnology, Hunan, China). The relative quantities of enrolled genes normalized to the internal control were calculated using the method of 2^–ΔΔCt^.

### Enzyme assays and kinetics

We measured the enzymatic activities of IDH (EC 1.1.1.42), α-KGDH (EC 1.2.4.2), SCS (EC 6.2.1.5), and SDH (EC 1.3.5.1), the key enzymes of the TCA pathway, derived from *L. salsilacus* grown at 5 °C or 25 °C. Cells were cultivated in marine broth 2216 to the early stationary phase at 5 °C or 25 °C before being pelleted by centrifugation (5000 × *g* for 5 min, 4 °C) for protein extraction. The obtained pellets were resuspended and lysed with BugBuster Master Mix (Novagen, Darmstadt, Germany) according to the manufacturer’s protocol, and the protein concentrations of the obtained extracts were determined using the Bicinchoninic Acid Protein Assay Kit (Takara, Shiga, Japan). For quantified assays, 0.2 μg of protein was added and the enzymatic activities of IDH, α-KGDH, and SCS were both measured at 5 °C and 25 °C using Isocitrate Dehydrogenase Activity, α-Ketoglutarate Dehydrogenase, and Succinyl-CoA Synthetase Activity Assay Kits (Sigma-Aldrich, SL, USA) following the manufacturer’s instructions. 20 μg protein was used to measure the activity of SDH by Micro Succinate Dehydrogenase Assay Kit (Solarbio Science & Technology, Beijing, China) at 5 °C and 25 °C following the manufacturer’s instructions. Variations of the absorbance were continuously detected with a microplate reader (Cytation 5, BioTek, VT, USA) using the kinetic model. One unit of IDH was defined as the amount of enzyme that generates 1.0 μmole of NADPH per minute at pH 8.0 at 5 °C or 25 °C, one unit of α-KGDH was defined as the amount of enzyme that generates 1.0 μmole of NADH per minute at pH 7.5 at 5 °C or 25 °C, one unit of SCS was defined as the amount of enzyme that generates 1.0 mmole of NADH per minute at pH 7.4 at 5 °C or 25 °C, and one unit of SDH was defined as the amount of enzyme that generates 1.0 μmole of DCIP per minute at pH 7.2 at 5 °C or 25 °C.

### Carbon source consumption experiments

To measure the activity of the TCA cycle and pattern of carbohydrate utilization during cold acclimation, growth tests of *L. salsilacus* were carried out as described below. Briefly, strains cultured in marine broth 2216 at 5 °C or 25 °C were used as the first precultures, and the cell pellets were harvested by centrifugation (5000 × *g* for 5 min), washed three times with sterile seawater prepared with 30 g/L sea salts (Sigma-Aldrich), starved for 2 h in sterile seawater at 5 °C or 25 °C, placed in a 120 rpm shaker, and used as the inoculum for the defined medium in triplicate. The sole carbon source medium from Chen et al.^70^ was improved for the study and contained 30 g/L sea salts, 10 mM (pH 8.0) HEPES (Sigma-Aldrich), 3 mM ammonium chloride (Sigma-Aldrich), 0.2 mM sodium phosphate (Sigma-Aldrich), 50 μM FeCl_3_ (MDBio, Taiwan, China), and a mixture of vitamins. Additionally, a series of sole carbon sources were supplemented with 20 mM (pH 8.0) citric acid (Sigma-Aldrich) or 20 mM (pH 8.0) succinate (MDBio) to test for TCA cycle substrates; 20 mM glucose (SCRC, Shanghai, China), 20 mM galactose (SCRC), 20 mM fructose (SCRC), 20 mM rhamnose (SCRC), 20 mM xylose (SCRC), 10 mM sucrose (SCRC), 10 mM maltose (SCRC), 10 mM cellobiose (SCRC), 2 g/L soluble starch (Yuanye, Shanghai, China), or 2 g/L xylan (Megazyme, Ireland) representing different classes of carbohydrates that are ubiquitous in polar regions. Cultures were incubated at 5 °C or 25 °C on a 180 rpm shaker, and growth performances were validated by maximum biomass represented by OD_600_, as determined with a spectrophotometer. Differences in the maximum biomass between two temperatures were statistically evaluated for significance by *t*-test using GraphPad Prism.

For the mixed carbon sources test, *L. salsilacus* strains were grown in the medium supplied with both 10 mM glucose and sucrose, and other conditions were the same as those used in the sole carbon source experiment. The consumption of carbon sources in the medium was continuously monitored with a Glucose Assay Kit and Sucrose Assay Kit (Solarbio Life Sciences, Beijing, China) following the manufacturer’s protocol until the concentrations of glucose and sucrose in the medium showed no obvious variation. Finally, the depletion of glucose and sucrose at maximum OD_600_ and during the stationary phase was measured.

### Statistics and reproducibility

For data analyses, two-tailed *t*-test was applied in all cases and *p*-value <0.05 is considered statically significant. The data were performed as the mean ± standard deviation, and the sample size for each experimental condition was given in the data dot plots. All experiments were repeated at least three times. The reproducibility of these experiments is well.

### Reporting summary

Further information on research design is available in the Nature Research Reporting Summary linked to this article.

## Supplementary information

Supplementary Information

Reporting Summary

## Data Availability

The source data for generating main figures are deposited in 10.6084/m9.figshare.19669572^71^. Genome and transcriptome data generated in this study are deposited in the National Center for Biotechnology Information (NCBI) under the BioProject accession number PRJNA721246. The complete genome of *L. salsilacus* 1A07893 is available under CP072991-CP072993 in the NCBI GenBank database, and *L. salsilacus* 1A07899 is CP072994-CP072995. Raw reads data of transcriptome of *L. salsilacus* 1A07893 at different temperatures are available under SRR15255042-SRR15255056 in the NCBI in the Sequence Read Archive (SRA) database, and *L. salsilacus* 1A07899 is SRR15257723-SRR15257737.
