# Supplementary material for: TCA cycle enhancement and uptake of monomeric substrates support growth of marine Roseobacter at low temperature
Source: Commun Biol. 2022 Jul 14;5:705. doi: 10.1038/s42003-022-03631-2 (PMC9283371; doi:10.1038/s42003-022-03631-2)
Supplement: Supplementary file 1 — Supplementary Information [file 42003_2022_3631_MOESM1_ESM.pdf]

Supplementary Information for

TCA cycle enhancement and uptake of monomeric substrates support growth of marine *Roseobacter* at low temperature

Meng Wang[1,2,3], Huan Wang[1], Peng Wang[1], Hui-Hui Fu[1], Chun-Yang Li[1], Qi-Long, Qin[1,3], Yantao Liang[1], Min Wang[1], Xiu-Lan Chen[3,4], Yu-Zhong Zhang[1,2,3,4]*, Weipeng Zhang[1]*

[1]College of Marine Life Sciences, Ocean University of China, Qingdao, 266003, China

[2]Frontiers Science Center for Deep Ocean Multispheres and Earth System, Ocean University of China, Qingdao, 266003, China

[3]State Key Laboratory of Microbial Technology, Marine Biotechnology Research Center, Shandong University, Qingdao, 266237, China

[4]Laboratory for Marine Biology and Biotechnology, Pilot National Laboratory for Marine Science and Technology, Qingdao, 266373, China

*Correspondence to Yu-Zhong Zhang (zhangyz@sdu.edu.cn) or Weipeng Zhang (zhangweipeng@ouc.edu.cn)

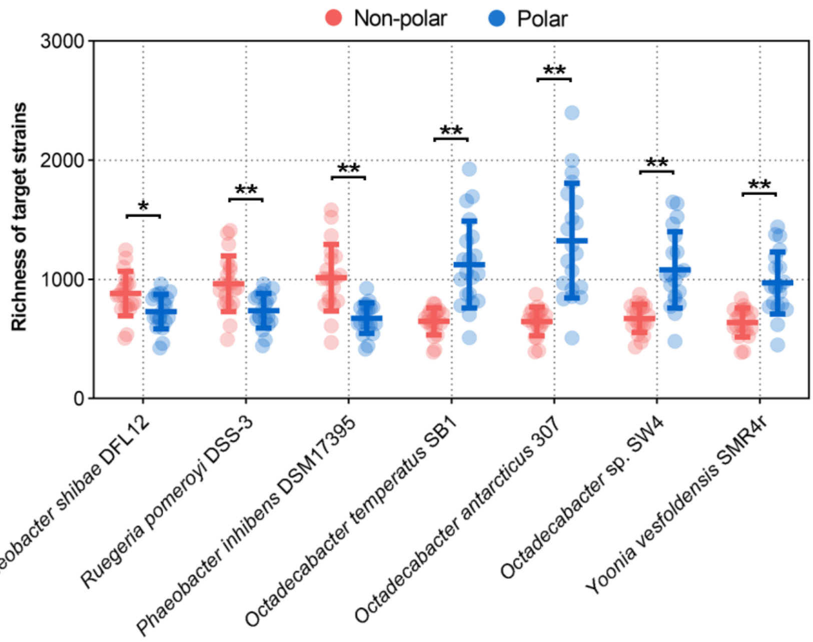

Supplementary Fig. 1 Abundances of typical *Roseobacter* group bacteria in polar and non-polar regions. Abundances of *Roseobacter* group bacteria (RGB) recovered from 40 global seawater metagenomes, including those from polar and non-polar regions, are shown. Differences between the average abundance of RGB in non-polar and polar regions were estimated by a two-tailed *t*-test, and significant differences were indicated by asterisks (* p <0.05; ** p <0.005). The detailed information of these isolates is mentioned in Supplementary Table 1 below.

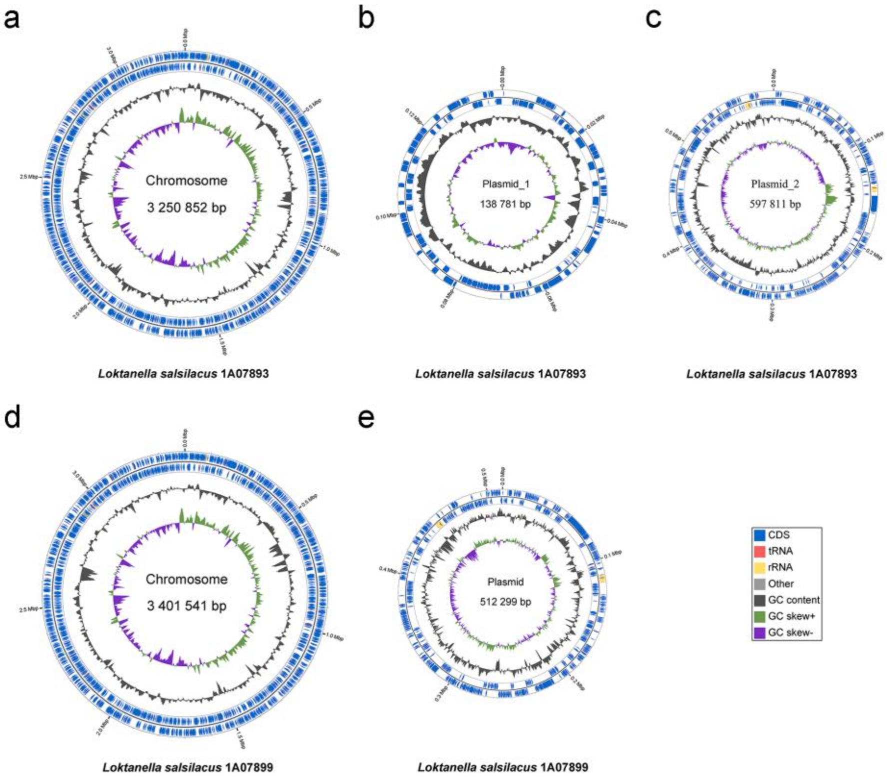

Supplementary Fig. 2 The circular genomes of *L. salsilacus* 1A07893 and 1A07899. Each plot represents a chromosome or a plasmid. Rings from the outer to inner indicate: 1) predicted protein-coding sequences (blue), tRNA (red), and rRNA (yellow) genes on forward or reverse strands; 2) GC content ratios compared with the average GC content of the whole genome; 3) GC skew across the whole genome (green, positive; purple, negative).

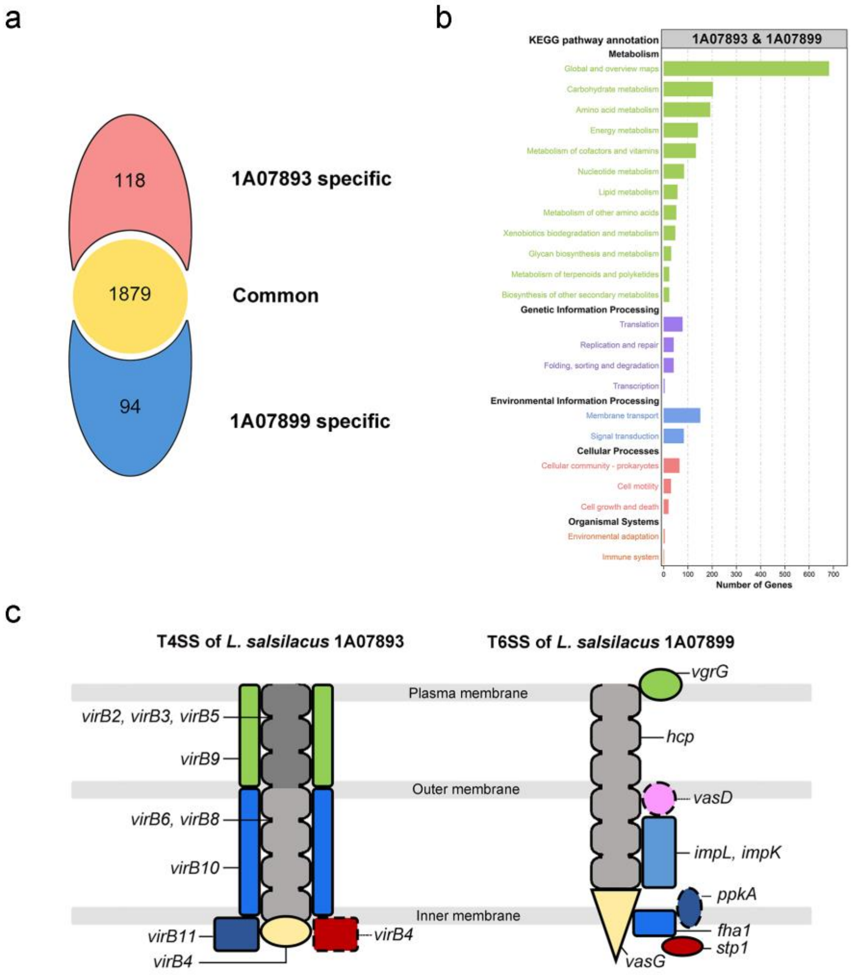

Supplementary Fig. 3 Comparison of KEGGs between *L. salsilacus* 1A07893 and 1A07899. a A venn diagram to shew the specific and common KEGGs between 1A07893 and 1A07899. **b** KEGG level 2 classification of the KEGGs shared by 1A07893 and 1A07899. **c** Different bacterial secretion systems carried by *L. salsilacus* 1A07893 and 1A07899. Solid lines represent components that are present in the genomes, while dotted lines represent genes absent in the genomes.

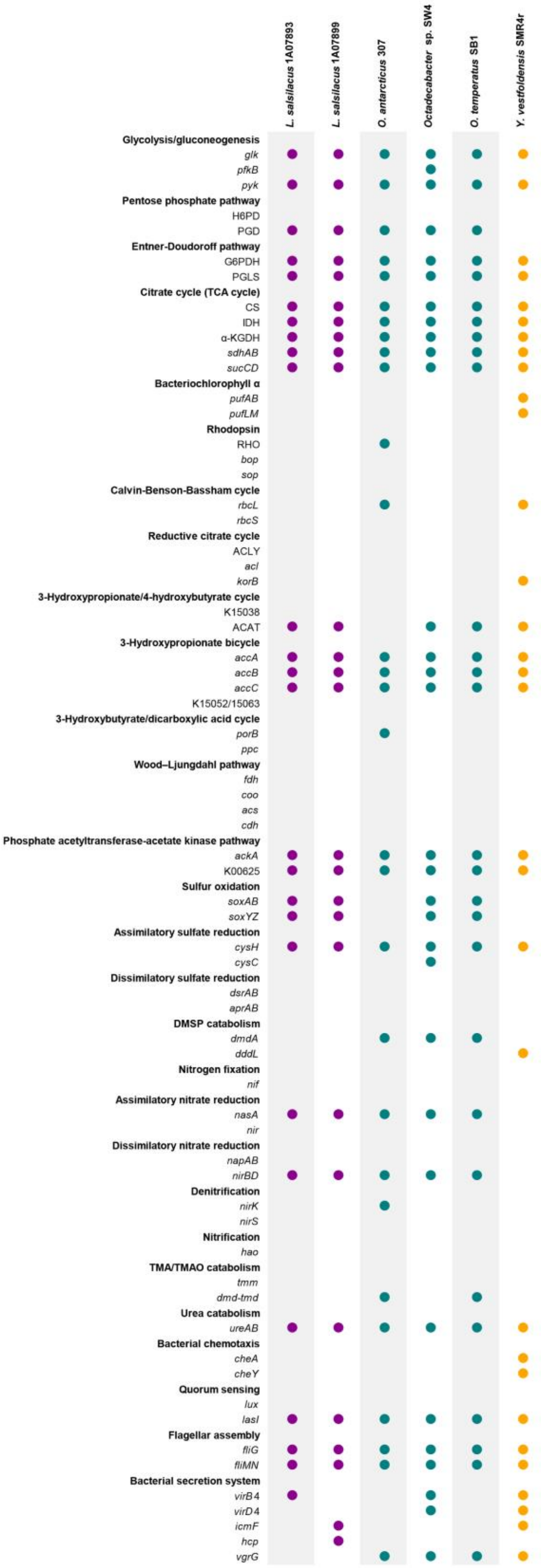

Supplementary Fig. 4 Comparative analyses of potential metabolic abilities of *L. salsilacus* and closely related RGB strains. A matrix with dots indicates the presence of core genes related to key metabolic pathways, and strains that belong to different genera are labeled with different colors (purple, *Loktanella*; green, *Octadecabacter*; yellow, *Yoonia*).

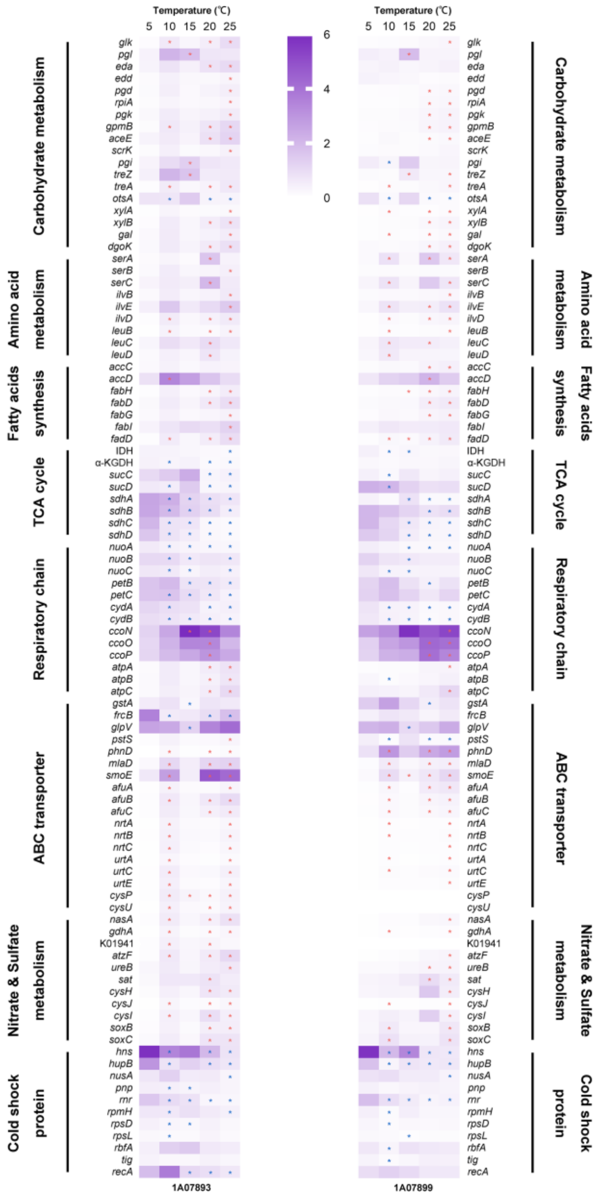

Supplementary Fig. 5 Differentially transcribed genes in *L. salsilacus* when grown at different temperatures. Pairwise comparisons of 5°C vs. 10°C, 5°C vs. 15°C, 5°C vs. 20°C, and 5°C vs. 25°C were performed. The genes with significant differences in at least one comparison are plotted in the heatmaps. The color matrix reflects the scaled reads per kilobase per million mapped reads (RPKMs) of a given gene. The average values of the three replicates are weighted. Differentially expressed genes are identified when *p*-value <0.05 with fold-change of RPKMs >2 and labeled by asterisks. Red asterisks represent upregulated genes at 10°C, 15°C, 20°C, and 25°C compared to 5°C. Blue asterisks indicate upregulated genes at 5°C compared to the current temperature.

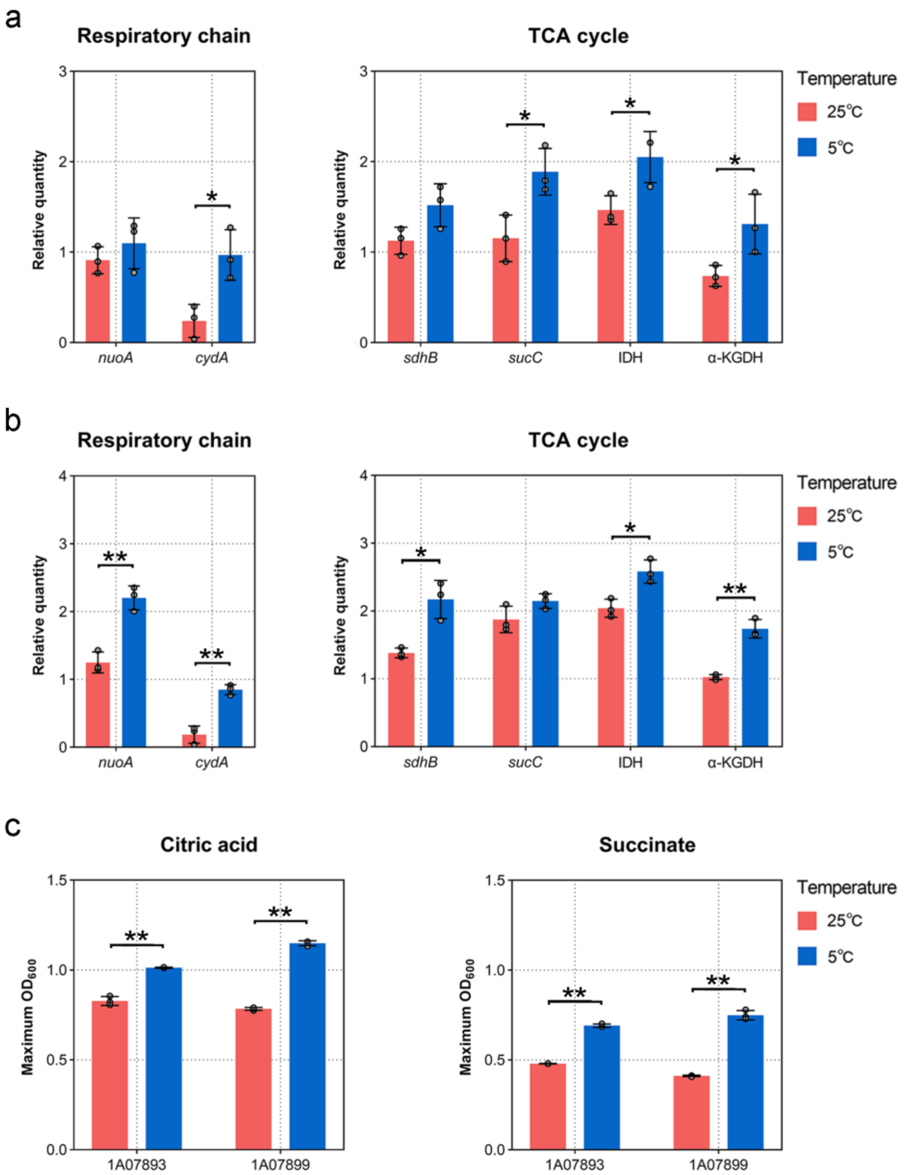

Supplementary Fig. 6 Validation of the transcriptomic results by RT-qPCR and growth test. a, b Transcription levels of TCA- and respiration-related genes of *L. salsilacus* 1A07893 (**a**) and 1A07899 (**b**) at 5℃ and 25℃, as determined by RT-qPCR. The relative quantity of a given gene was determined using the method of $2^{-\Delta\Delta Ct}$ and normalized by $\log_{10}$. **c** Maximum growth OD_{600} of the two strains using TCA intermediates as the sole carbon source. Error bars represent standard deviations and differences are statistically evaluated for significance by asterisks (* p <0.05; ** p <0.005).

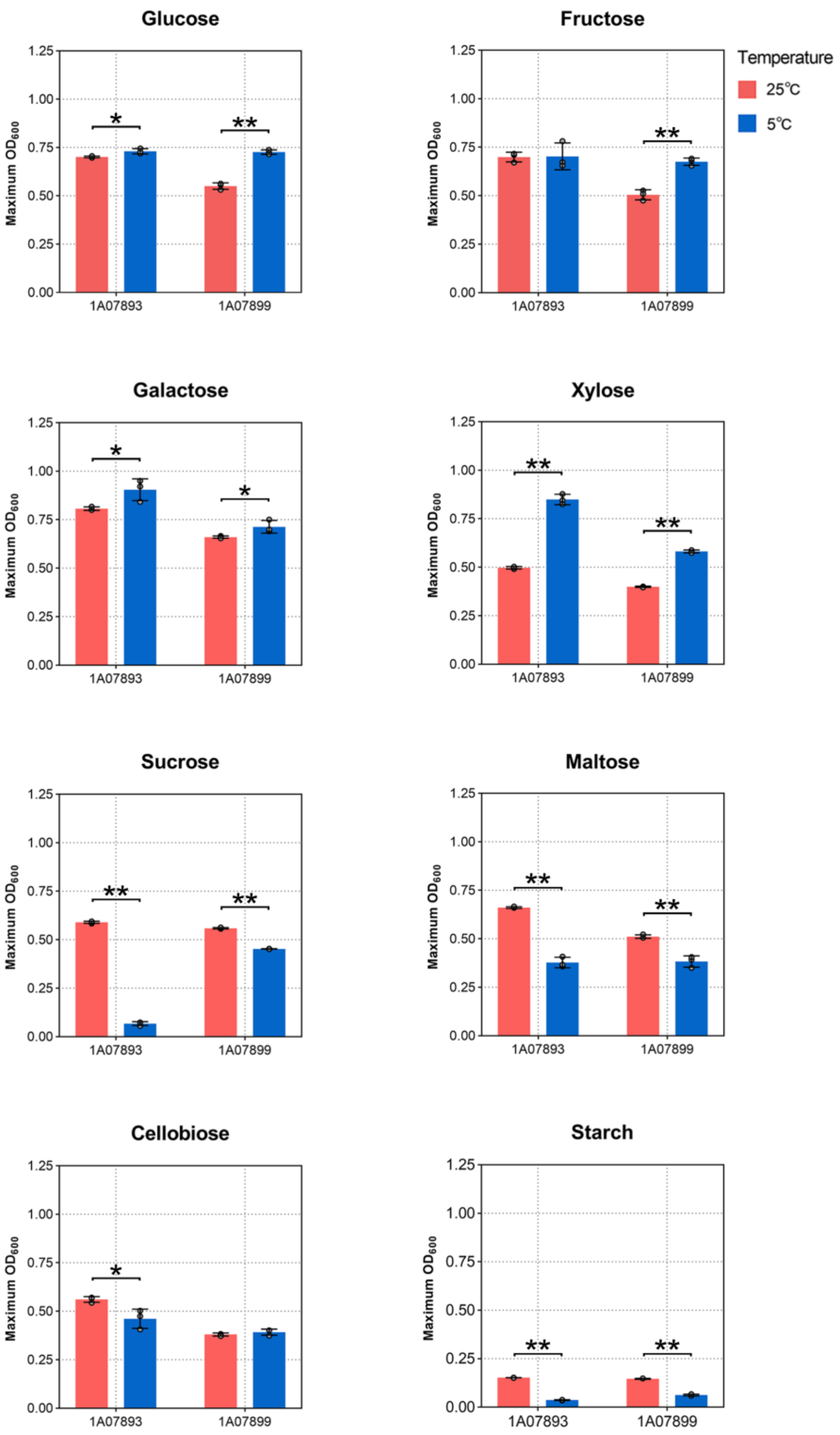

Supplementary Fig. 7 Preferential utilization of carbohydrates by *L. salsilacus* when grown at different temperatures. Capabilities of *L. salsilacus* in utilizing different carbohydrates were indicated by maximum growth biomass. Carbohydrates accounting for the majority of the dissolved organic carbon pools in marine environments were selected for the experiments, which were performed in triplicates and the average values are given. Error bars represent standard deviations and differences are statistically evaluated for significance by asterisks (* p <0.05; ** p <0.005).

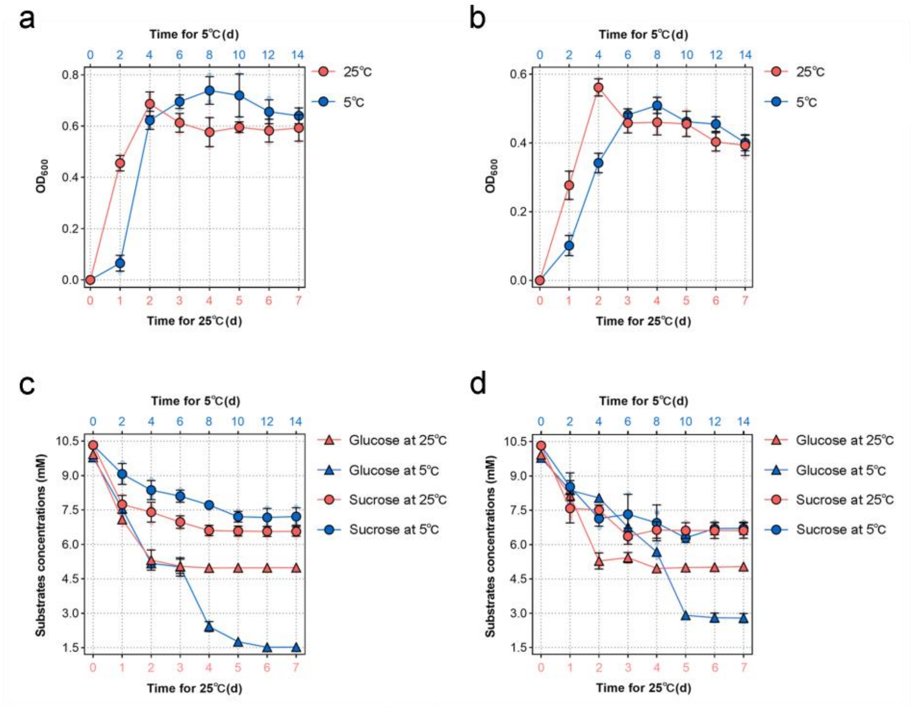

Supplementary Fig. 8 Biomass and substrates depletion of *L. salsilacus* grown on the defined medium at different temperatures. Concentrations of glucose and sucrose in the media, as well as cell biomass (OD_{600}), were measured at a series of time points after seeding of the bacterial strains (**a** 1A07893 and **b** 1A07899). The experiment was performed in triplicate, and the average values are given. Error bars represent standard deviations.

Supplementary Table 1 Growth temperature profiles of *L. salsilacus* and typical RGB members.

Strains	Growth temperature range	Optimal growth temperature	Optimum growth rate (h^{-1})	Growth medium
Loktanella salsilacus 1A07893	0-30°C	25°C	0.06	Marine broth 2216
Loktanella salsilacus 1A07899	0-30°C	25°C	0.08	Marine broth 2216
Dinoroseobacter shibae DFL 12	15-38°C	33°C	0.17	A complex medium containing sea salts, peptone and yeast extract
Ruegeria pomeroyi DSS-3	10-40°C	30°C	0.02	Marine basal medium
Phaeobacter inhibens DSM17395	15-37°C	23-27°C	0.36	Marine broth 2216
Octadecabacter antarcticus 307	4-10°C	NP	NP	Sea Water Cytophaga medium
Octadecabacter sp. SW4	NP	NP	NP	NP
Octadecabacter temperatus SB1	4-25°C	20°C	NP	Marine broth with slight modifications
Yoonia vestfoldensis SMR4r	NP	NP	NP	NP

Abbreviations: NP, not published.

Supplementary Table 2 Information of the metagenomes included for bacterial distribution analyses.

ENA_ID	Station location					Abundance (per million reads)	
	Longitude	Latitude	Depth, nominal	Waters	Polar or not	1A07893	1A07899
ERS488330	14.26 E	35.76 N	5	[MS] Mediterranean Sea	Non polar	646	647
ERS488551	34.84 E	27.16 N	5	[RS] Red Sea	Non polar	766	775
ERS488621	38.25 E	21.95 N	5	[RS] Red Sea	Non polar	710	710
ERS489043	69.98 E	14.61 N	5	[IO] Indian Ocean	Non polar	528	531
ERS489087	73.90 E	6.00 N	5	[IO] Indian Ocean	Non polar	525	527
ERS489529	53.98 E	16.96 S	5	[IO] Indian Ocean	Non polar	659	658
ERS490327	3.18 W	20.41 S	5	[SAO] South Atlantic Ocean	Non polar	395	399
ERS490433	17.91 W	8.78 S	5	[SAO] South Atlantic Ocean	Non polar	420	419
ERS491836	95.98 W	13.00 S	5	[SPO] South Pacific Ocean	Non polar	706	713
ERS492321	100.63 W	16.96 S	5	[SPO] South Pacific Ocean	Non polar	599	599
ERS492642	139.2 W	9.00 S	5	[SPO] South Pacific Ocean	Non polar	749	758
ERS493044	153.68 W	0	5	[SPO] South Pacific Ocean	Non polar	741	748
ERS493300	159.00 W	31.52 N	5	[NPO] North Pacific Ocean	Non polar	749	749
ERS493390	127.74 W	35.37 N	5	[NPO] North Pacific Ocean	Non polar	817	821
ERS493636	116.63 W	14.2 N	5	[NPO] North Pacific Ocean	Non polar	610	608
ERS493752	102.94 W	6.33 N	5	[NPO] North Pacific Ocean	Non polar	749	758
ERS493938	88.39 W	25.53 N	5	[NAO] North Atlantic Ocean	Non polar	748	741
ERS494394	49.92 W	34.11 N	5	[NAO] North Atlantic Ocean	Non polar	612	612
ERS494445	37.30 W	35.93 N	5	[NAO] North Atlantic Ocean	Non polar	631	628
ERS494518	29.02 W	36.17 N	5	[NAO] North Atlantic Ocean	Non polar	609	609
ERS1307873	16.94 W	54.53 N	5	[NAO] North Atlantic Ocean	Polar	809	807
ERS1307987	0.24 E	67.14 N	5	[AO] Arctic Ocean	Polar	754	768
ERS1308483	1.39 E	76.18 N	5	[AO] Arctic Ocean	Polar	1441	1466
ERS1308550	44.08 E	72.51 N	5	[AO] Arctic Ocean	Polar	990	995
ERS1308688	79.42 E	78.96 N	5	[AO] Arctic Ocean	Polar	1371	1410
ERS1308760	66.34 E	79.22 N	5	[AO] Arctic Ocean	Polar	1123	1162
ERS1308875	73.21 E	77.16 N	5	[AO] Arctic Ocean	Polar	1529	1592
ERS1308921	76.15 E	74.80 N	5	[AO] Arctic Ocean	Polar	1346	1394
ERS1309018	91.86 E	78.25 N	5	[AO] Arctic Ocean	Polar	972	979
ERS1309098	117.15 E	77.90 N	5	[AO] Arctic Ocean	Polar	1151	1168
ERS1309205	160.94 E	71.60 N	5	[AO] Arctic Ocean	Polar	651	668
ERS1309247	174.99 E	71.07 N	5	[AO] Arctic Ocean	Polar	1229	1245
ERS1309291	168.13 W	73.38 N	5	[AO] Arctic Ocean	Polar	1009	1010
ERS1309379	154.91 W	71.89 N	5	[AO] Arctic Ocean	Polar	932	951
ERS1309458	85.78 W	74.30 N	5	[AO] Arctic Ocean	Polar	994	1013
ERS1309543	71.89 W	72.47 N	5	[AO] Arctic Ocean	Polar	1169	1196
ERS1309625	53.6 W	70.96 N	5	[AO] Arctic Ocean	Polar	782	810
ERS1309696	51.51 W	69.11 N	5	[AO] Arctic Ocean	Polar	487	490
ERS1309745	53.01 W	64.71 N	5	[AO] Arctic Ocean	Polar	811	825
ERS1309819	55.99 W	61.54 N	5	[AO] Arctic Ocean	Polar	774	789

Supplementary Table 3 General features of the complete genomes of *L. salsilacus* 1A07893 and 1A07899.

Characteristic	*L. salsilacus* 1A07893			*L. salsilacus* 1A07899	
	Chromosome	Plasmid_1	Plasmid_2	Chromosome	Plasmid
Size (bp)	3,250,852	138,781	597,811	3,401,541	512,299
GC percentage (%)	60.55	56.49	57.82	60.33	58.2
Predicted CDSs (n)	3,140	139	539	3,275	445
Avg. size of CDS (bp)	940	830	980	938	1,000
Annotated CDSs (n)	2,414	78	418	2,460	337
rRNAs (n)	6	0	6	6	6
tRNAs (n)	46	0	8	46	8

Supplementary Table 4 Complete RGB genomes used for comparative analyses with *L. salsilacus*.

Microorganism	Genome size (Mb)	GenBank accession number	References
Dinoroseobacter shibae DFL 12	4.42	NC_009952	NP
Jannaschia sp. CCS1	4.40	NC_007802	[1]
Ketogulonicigenium robustum SPU_B003	2.71	NZ_CP019937	[2]
Ketogulonicigenium vulgare SKV	3.03	NZ_CP016592	[3]
Ketogulonigenium vulgarum WSH-001	3.28	NC_017384	[4]
Leisingera methylohalidivorans DSM 14336	4.65	NC_023135	[5]
Leisingera sp. NJS201	5.14	NZ_CP038234	NP
Maribius sp. THAF1	3.34	NZ_CP045420	NP
Octadecabacter antarcticus 307	4.88	NC_020911	[6]
Octadecabacter sp. SW4	3.54	NZ_CP042819	NP
Octadecabacter temperatus SB1	3.26	NZ_CP012160	[7]
Phaeobacter gallaeciensis DSM 26640	4.54	NC_023137	NP
Phaeobacter inhibens 2.10	4.16	NC_018286	[8]
Phaeobacter inhibens DSM 17395	4.23	NC_018290	[8]
Phaeobacter piscinae P71	4.08	NZ_CP010656	[9]
Phaeobacter porticola P97	4.21	NZ_CP016364	[10]
Rhodobacter capsulatus SB 1003	3.87	NC_014034	[11]
Rhodobacter sphaeroides 2.4.1	4.63	NZ_CP030271	NP
Rhodobacter sphaeroides MBTLJ-20	4.65	NZ_CP015287	NP
Rhodobacteraceae bacterium G7	3.34	CP021114	NP
Roseobacter denitrificans OCh 114	4.33	NC_008209	[12]
Roseobacter litoralis Och149	4.75	NC_015730	[13]
Roseovarius indicus DSM 26383	6.17	NZ_CP031598	NP
Roseovarius mucosus SMR3	4.38	NZ_CP020474	[14]
Roseovarius sp. AK1035	4.20	NZ_CP030099	NP
Ruegeria pomeroyi DSS-3	4.60	NC_003911	[15]
Ruegeria sp. TM1040	4.15	NC_008044	[1]
Sulfitobacter sp. AM1-D1	4.69	NZ_CP018076	[16]
Sulfitobacter sp. THAF37	4.26	NZ_CP045372	NP
Thalassococcus sp. S3	5.00	NZ_CP022303	[17]
Yoonia vestfoldensis SMR4r	3.99	NZ_CP021431	[18]

Abbreviations: NP, not published.

Supplementary Table 5 Primers for RT-qPCR experiments.

Gene	Forward primer	Reverse primer	Product size (bp)
rplC	CAAGGCGAATGTGGAACCAA	AGTGATTTCTTCGCCAACGG	85
nuoA	TCAGCTTATGAATGCGGCTT	ACACGAGGTAGAATCGCACA	73
cydA	CCAATTCGCGTTCACCATCT	ACGATGCGGTATGCATTGTC	123
sucC	TGGTCAAGGCACAAATCCAC	GGCTTCTTCAACCGACTTGG	107
sdhB	TAGATGGTCGCTACGAGTGC	AAGGTAGCGGTCAGAGTTCC	83
IDH	AGGTAGCTAACCCGATCGTC	TACAGCAGATCCACGTCCAG	109
KGDH	GACCGGGACAAGGACAACTA	CGTCTTGTGAGTCGCGAAAT	114

Supplementary references

1. Moran, MA. et al. Ecological genomics of marine roseobacters. *Appl. Environ. Microbiol.* **73**, 4559-4569 (2007).
2. Wang, CY. et al. Establishing an innovative carbohydrate metabolic pathway for efficient production of 2-keto-L-gulonic acid in *Ketogulonicigenium robustum* initiated by intronic promoters. *Microb. Cell. Fact.* **17**, 81 (2018).
3. Jia, N., Ding, MZ., Du, YZ., Feng, S., Gao, F. & Yuan, YJ. Complete genome sequence of the industrial bacterium *Ketogulonicigenium vulgare* SKV. *Genome. Announc.* **4**, e01426-16 (2016).
4. Liu, L. et al. Complete genome sequence of the industrial strain *Ketogulonicigenium vulgare* WSH-001. *J. Bacteriol.* **193**, 6108-6109 (2011).
5. Buddruhs, N. et al. Complete genome sequence of the marine methyl-halide oxidizing *Leisingera methylohalidivorans* type strain (DSM 14336(T)), a representative of the *Roseobacter* clade. *Stand. Genomic. Sci.* **9**, 128-141 (2013).
6. Vollmers, J. et al. Poles apart: Arctic and Antarctic *Octadecabacter* strains share high genome plasticity and a new type of xanthorhodopsin. *Plos One* **8**, e63422 (2013).
7. Voget, S., Billerbeck, S., Simon, M. & Daniel, R. Closed genome sequence of *Octadecabacter temperatus* SB1, the first mesophilic species of the genus *Octadecabacter*. *Genome. Announc.* **3**, e01051-15 (2015).
8. Thole, S. et al. *Phaeobacter gallaeciensis* genomes from globally opposite locations reveal high similarity of adaptation to surface life. *ISME. J.* **6**, 2229-2244 (2012).
9. Freese, HM. et al. Trajectories and drivers of genome evolution in surface-associated marine *Phaeobacter*. *Genome. Biol. Evol.* **9**, 3297-3311 (2017).
10. Breider, S., Freese, HM., Sproer, C., Simon, M., Overmann, J. & Brinkhoff, T. *Phaeobacter porticola* sp. nov., an antibiotic-producing bacterium isolated from a sea harbour. *Int. J. Syst. Evol. Microbiol.* **67**, 2153-2159 (2017).
11. Strnad, H. et al. Complete genome sequence of the photosynthetic purple nonsulfur bacterium

Rhodobacter capsulatus SB 1003. *J. Bacteriol.* **192**, 3545-3546 (2010).

12. Swingley, WD. et al. The complete genome sequence of *Roseobacter denitrificans* reveals a mixotrophic rather than photosynthetic metabolism. *J. Bacteriol.* **189**, 683-690 (2007).
13. Kalhoefer, D. et al. Comparative genome analysis and genome-guided physiological analysis of *Roseobacter litoralis*. *BMC Genomics* **12**, 324 (2011).
14. Topel, M., Pinder, MIM., Johansson, ON., Kourtchenko, O., Godhe, A. & Clarke, AK. Genome sequence of *Roseovarius mucosus* strain SMR3, isolated from a culture of the diatom *Skeletonema marinoi*. *Genome. Announc.* **5**, e00394-17 (2017).
15. Moran, MA. et al. Genome sequence of *Silicibacter pomeroyi* reveals adaptations to the marine environment. *Nature* **432**, 910-913 (2004).
16. Yang, X. et al. Complete genome sequence of a toxic and bioactive exopolysaccharide-bearing bacterium, *Sulfitobacter* sp. strain AM1-D1. *Microbiol. Resour. Announc.* **9**, e00232-20 (2020).
17. Vejarano, F., Suzuki-Minakuchi, C., Ohtsubo, Y., Tsuda, M., Okada, K. & Nojiri, H. Complete genome sequence of *Thalassococcus* sp. strain S3, a marine *Roseobacter* clade member capable of degrading carbazole. *Microbiol. Resour. Announc.* **8**, e00231-19 (2019).
18. Topel, M., Pinder, MIM., Johansson, ON., Kourtchenko, O., Godhe, A. & Clarke, AK. Complete genome dequence of *Loktanella vestfoldensis* strain SMR4r, a novel strain isolated from a culture of the chain-forming diatom *Skeletonema marinoi*. *Genome. Announc.* **6**, e01558-17 (2018).
