# Supplementary material for: TCA cycle enhancement and uptake of monomeric substrates support growth of marine Roseobacter at low temperature
Source: Commun Biol. 2022 Jul 14;5:705. doi: 10.1038/s42003-022-03631-2 (PMC9283371; doi:10.1038/s42003-022-03631-2)
Supplement: Supplementary file 2 — Reporting Summary [file 42003_2022_3631_MOESM2_ESM.pdf]

nature portfolio

Corresponding author(s): Weipeng Zhang

Last updated by author(s): Nov 22, 2021

Reporting Summary

Nature Portfolio wishes to improve the reproducibility of the work that we publish. This form provides structure for consistency and transparency in reporting. For further information on Nature Portfolio policies, see our Editorial Policies and the Editorial Policy Checklist.

Statistics

For all statistical analyses, confirm that the following items are present in the figure legend, table legend, main text, or Methods section.

n/a	Confirmed	
☐	☒	The exact sample size (n) for each experimental group/condition, given as a discrete number and unit of measurement
☐	☒	A statement on whether measurements were taken from distinct samples or whether the same sample was measured repeatedly
☐	☒	The statistical test(s) used AND whether they are one- or two-sided<br>*Only common tests should be described solely by name; describe more complex techniques in the Methods section.*
☒	☐	A description of all covariates tested
☒	☐	A description of any assumptions or corrections, such as tests of normality and adjustment for multiple comparisons
☒	☐	A full description of the statistical parameters including central tendency (e.g. means) or other basic estimates (e.g. regression coefficient) AND variation (e.g. standard deviation) or associated estimates of uncertainty (e.g. confidence intervals)
☒	☐	For null hypothesis testing, the test statistic (e.g. F, t, r) with confidence intervals, effect sizes, degrees of freedom and P value noted<br>*Give P values as exact values whenever suitable.*
☒	☐	For Bayesian analysis, information on the choice of priors and Markov chain Monte Carlo settings
☒	☐	For hierarchical and complex designs, identification of the appropriate level for tests and full reporting of outcomes
☒	☐	Estimates of effect sizes (e.g. Cohen's d, Pearson's r), indicating how they were calculated

Our web collection on statistics for biologists contains articles on many of the points above.

Software and code

Policy information about availability of computer code

Data collection	Genomic data were collected by Loktanella salsilacus strains purchased from Marine Culture Collection of China and DNA sequencing on the PacBio Sequel and Illumina NovaSeq platform. Transcriptomic data were collected by total RNA extracted from L. salsilacus grown at different temperatures and RNA-seq on the Illumina Novaseq platform.
Data analysis	All the software and scripts used in this manuscript are publicly available or published in previous studies. All the related references are listed after the software in the Method part of the main text. The software used in this manuscript includes DMfit (version 3.5), SMRT Link (version 5.0.1), NGS QC Toolkit (version 2.3.3), SeqKit (version 0.15.0), BBMap (version 38.87), Prokka (version 1.14.6), AMPHORA2, MEGA6, Ggtree (version 3.12), Bowtie2 (version 2.4.2), Samtools (version 1.11).

For manuscripts utilizing custom algorithms or software that are central to the research but not yet described in published literature, software must be made available to editors and reviewers. We strongly encourage code deposition in a community repository (e.g. GitHub). See the Nature Portfolio guidelines for submitting code & software for further information.

Data

Policy information about availability of data

All manuscripts must include a data availability statement. This statement should provide the following information, where applicable:

- Accession codes, unique identifiers, or web links for publicly available datasets
- A description of any restrictions on data availability
- For clinical datasets or third party data, please ensure that the statement adheres to our policy

The source data for generating main figures are deposited in https://doi.org/10.6084/m9.figshare.19669572. Genome and transcriptome data generated in this study have been deposited the National Center for Biotechnology Information (NCBI) under the BioProject accession number PRJNA721246. The complete genome

of L. salsilacus 1A07893 is available under CP072991-CP072993 in the NCBI GenBank database, and L. salsilacus 1A07899 is CP072994-CP072995. Raw reads data of transcriptome of L. salsilacus 1A07893 at different temperature are available under SRR15255042-SRR15255056 in the NCBI in the Sequence Read Archive database, and L. salsilacus 1A07899 is SRR15257737-SRR15257741.

Field-specific reporting

Please select the one below that is the best fit for your research. If you are not sure, read the appropriate sections before making your selection.

☒ Life sciences ☐ Behavioural & social sciences ☐ Ecological, evolutionary & environmental sciences

For a reference copy of the document with all sections, see nature.com/documents/nr-reporting-summary-flat.pdf

Life sciences study design

All studies must disclose on these points even when the disclosure is negative.

Sample size	The two L. salsilacus strains were enrolled in this research, and their complete genomes of strains (n=2) and transcriptomic data generated at five different temperatures (n=30) were included for analyses.
Data exclusions	No data were excluded.
Replication	The related files, including intermediate results required to reproduce the results presented in the manuscript, are provided in the Data availability part of the manuscript.
Randomization	Not applicable.
Blinding	Not applicable.

Reporting for specific materials, systems and methods

We require information from authors about some types of materials, experimental systems and methods used in many studies. Here, indicate whether each material, system or method listed is relevant to your study. If you are not sure if a list item applies to your research, read the appropriate section before selecting a response.

Materials & experimental systems

n/a	Involved in the study	
☒	☐	Antibodies
☒	☐	Eukaryotic cell lines
☒	☐	Palaeontology and archaeology
☒	☐	Animals and other organisms
☒	☐	Human research participants
☒	☐	Clinical data
☒	☐	Dual use research of concern

Methods

n/a	Involved in the study	
☒	☐	ChIP-seq
☒	☐	Flow cytometry
☒	☐	MRI-based neuroimaging
